# Facile Preparation of Magnetic COF-on-COF for Rapid Adsorption and Determination of Sulforaphane from Cruciferous Vegetables

**DOI:** 10.3390/foods13030409

**Published:** 2024-01-26

**Authors:** Jie Zhou, Dan Xu, Jiayong Cao, Weiye Shi, Xuan Zhang, Huan Lin, Chen Yin, Lingyun Li, Donghui Xu, Guangyang Liu

**Affiliations:** 1 State Key Laboratory of Vegetable Biobreeding, Institute of Vegetables and Flowers, Chinese Academy of Agricultural Sciences, Key Laboratory of Vegetables Quality and Safety Control, Ministry of Agriculture and Rural Affairs of China, Beijing 100081, Chinalinhuan03@caas.cn (H.L.); xudonghui@caas.cn (D.X.); 2 College of Life Sciences, Yantai University, Yantai 264005, China; 3 Hebei Key Laboratory of Quality and Safety Analysis-Testing for Agro-Products and Food, Hebei North University, Zhangjiakou 075000, China; 4 Institute of Biological Science and Engineering, Hebei University of Science and Technology, Shijiazhuang 050018, China

**Keywords:** magnetic covalent organic frameworks, magnetic dispersive solid-phase extraction, sulforaphane, cruciferous vegetables, determination

## Abstract

Sulforaphane (SFN) is a natural isothiocyanate compound widely abundant in cruciferous vegetables with multiple bioactive functions. However, traditional analytical methods for the extraction and determination of SFN are cumbersome, time-consuming, and low sensitivity with large amounts of organic solvents. Herein, novel magnetic COF-on-COFs (MB-COFs) were fabricated using Fe_3_O_4_ as a magnetic core and COFs-1 grown with COFs-2 as a shell, and they were used as efficient adsorbents of magnetic dispersive solid-phase extraction for rapid quantification of SFN in cruciferous vegetables by combining with HPLC-MS/MS. At the optimal ratio of COFs-1 to COFs-2, MB-COFs had a spherical cluster-like structure and a rough surface, with a sufficient magnetic response for rapid magnetic separation (1 min). Due to the introduction of Fe_3_O_4_ and COFs-2, MB-COFs exhibited outstanding extraction efficiencies for SFN (92.5–97.3%), which was about 18–72% higher than that of the bare COFs. Moreover, MB-COFs showed good adsorption capacity (Q_m_ of 18.0 mg/g), rapid adsorption (5 min) and desorption (30 s) to SFN, and favorable reusability (≥7 cycles) by virtue of their unique hierarchical porous structure. The adsorption kinetic data were well fitted by the pseudo-second-order, Ritchie-second-order, intra-particle diffusion, and Elovich models, while the adsorption isotherm data were highly consistent with the Langmuir, Temkin, and Redlich–Peterson models. Finally, under the optimized conditions, the developed method showed a wide linear range (0.001–0.5 mg/L), high sensitivity (limits of quantification of 0.18–0.31 μg/L), satisfactory recoveries (82.2–96.2%) and precisions (1.8–7.9%), and a negligible matrix effect (0.82–0.97). Compared to previous methods, the proposed method is faster and more sensitive and significantly reduces the use of organic solvents, which can achieve the efficient detection of large-scale samples in practical scenarios. This work reveals the high practical potential of MB-COFs as adsorbents for efficient extraction and sensitive analysis of SFN in cruciferous vegetables.

## 1. Introduction

Sulforaphane (SFN), a natural isothiocyanate compound with several bioactive effects, is widely abundant in cruciferous vegetables (e.g., broccoli, cauliflower, radish, and cabbage). SFN is a secondary metabolite produced by the enzymatic hydrolysis of its glucosinolate precursor (glucoraphanin or 4-methylsulfinylbutyl glucosinolate) by endogenous myrosinase when the vegetable tissues are physically damaged by chopping, grinding, or crushing [1]. This promising phytochemical has attracted significant attention owing to its anticancer, anti-inflammatory, antioxidant, and antibacterial effects [2,3]. Due to the above health benefits of SFN, several analytical approaches have been reported for its quantification in cruciferous vegetables, such as high-performance liquid chromatography (HPLC) [4], HPLC–tandem mass spectrometry (HPLC-MS/MS) [5], and gas chromatography–mass spectrometry (GC-MS) [6]. However, GC-MS can easily lead to poor results due to the thermal degradation of SFN in the injection port, while HPLC has a long separation time, large matrix interference, poor selectivity, and low sensitivity for SFN analysis, although it is often used and the official ISO method (ISO 9167:2019) [7]. In comparison, HPLC-MS/MS has become the most widespread method for the detection of SFN in complicated matrices owing to its high specificity and sensitivity [8]. The official method (NY/T 3674-2020) issued by China also adopted HPLC-MS/MS for SFN determination in *Brassica napus* [9].

Sample pretreatment is required before the above instrumental analysis for the detection of SFN due to the disturbance of the complex vegetable matrix. Liquid–liquid extraction (LLE) is the most common pretreatment for SFN extraction from vegetables [10]. Nevertheless, this method usually needs repeated extraction with large amounts of organic solvents such as dichloromethane (DCM), ethyl acetate, chloroform, or methyl butyl ether, which is cumbersome, time-consuming, and environment-unfriendly. Compared to LLE, solid-phase extraction (SPE) has less organic solvent consumption and a strong cleaning and enrichment capacity [11]. However, the shortcomings of SPE are absorbent pretreatment prior to the extraction process and cartridge congestion, which makes it time-consuming and relatively expensive [12]. To overcome these drawbacks, magnetic dispersive solid-phase extraction (MSPE), as an ideal alternative approach, has been more widely proposed for sample pretreatment because of its easiness, rapidity, good recovery, short time, and economy. By incorporating the appealing surface features of non-magnetic adsorbents with magnetic nanoparticles, MSPE offers great potential in regard to its outstanding adsorption performance and fast phase separation. Obviously, the adsorbent plays the most crucial role in the MSPE procedure.

To date, several nanomaterials like metal oxides, graphene, multi-walled carbon nanotubes, mesoporous silica, layered double hydroxides (LDHs), polymer, metal–organic frameworks (MOFs), and covalent organic frameworks (COFs) have been developed as adsorbents for extracting target analytes in food samples [13,14,15]. Among them, COFs, as emerging organic porous adsorbents for the extraction of compounds in food, are composed of light-weight elements (i.e., C, H, O, N, and B) linked by strong covalent bonds, which have attracted a wide range of attention due to their tunable pore size, high specific surface areas, low density, good thermal and chemical stability, and easy modification of pore channels [16]. However, even after high-speed centrifugation, pure COFs are difficult to completely separate from the solution owing to their low density, resulting in a large mass loss of COFs and low extraction efficiency [17]. To solve this challenge and improve the functionality of COFs, magnetic COFs incorporating Fe-based magnetic cores have been gradually applied to the MSPE process, which eliminates the tedious centrifugation step and achieves the rapid and complete separation of COFs from the solution [18]. Nevertheless, most reported magnetic COFs are monolayer COFs, which interact with compounds mainly through weak interactions such as hydrogen bonding, hydrophobic interactions, π-π interactions, and van der Waals’ forces, resulting in low resistance to interference and poor extraction selectivity and efficiency for some analytes [19]. COF-on-COF is a heterogeneous structure consisting of two different COFs tightly bonded by strong chemical bonds, which have a staggered layered-sheet stacking structure with an interlaced pore network. Compared with monolayer COFs, COF-on-COFs are similar to a double-layer sieve with different pore sizes and shell thicknesses with excellent selective separation and catalytic performance [20,21]. Inspired by the above analysis, we developed a novel magnetic-based COF-on-COF (MB-COFs, i.e., Fe_3_O_4_@COF-on-COF) by growing a COFs-2 layer on the as-prepared Fe_3_O_4_@COFs-1 via the layer-by-layer assembly method to improve the adsorption performance. To the best of our knowledge, MB-COFs have not yet been prepared nor used as MSPE adsorbents for the extraction and determination of SFN in food samples.

In this work, MB-COFs were fabricated as efficient adsorbents of MSPE for the adsorption and determination of SFN from various SFN-rich vegetables by coupling with HPLC-MS/MS. The structural and morphological properties of MB-COFs were studied by various characterization techniques. The ratio of COFs-2/COFs-1 was varied to explore the effect on the extraction efficiency of SFN and the morphological structure of the final MB-COFs. After choosing the optimal MB-COFs, the MSPE parameters (including adsorbent amount, extraction time, pH, temperature, ionic strength, type and volume of desorption solvent, and desorption time) for SFN were systematically evaluated. The adsorption properties and mechanism of MB-COFs for SFN were also studied by various adsorption kinetic and isotherm models. Under optimized conditions, the validity of the proposed method was evaluated by linear range, coefficient of determination (R^2^), limit of detection (LOD), limit of quantification (LOQ), recovery, relative standard deviation (RSD), and matrix effect (ME). The aim of this work was to develop new MB-COFs and reveal their promising applications as an efficient adsorbent for the extraction and determination of SFN in complicated vegetable samples. Based on this, another aim was to establish a more rapid and sensitive method with less organic solvent usage to overcome the shortcomings of traditional methods and to achieve the efficient detection of large-scale samples in practical applications.

## 2. Materials and Methods

### 2.1. Chemicals and Materials

Ferric chloride hexahydrate (FeCl_3_⋅6H_2_O), ferrous sulfate heptahydrate (FeSO_4_·7H_2_O), polyethylene glycol 6000 (PEG), acetic acid (HAc), acetonitrile (ACN, HPLC grade), methanol (MeOH, HPLC grade), ammonium acetate (HPLC grade), dichloromethane (DCM), acetone (AC), and isopropanol (IPA) were obtained from Sinopharm Chemical Reagent Co., Ltd. (Shanghai, China). Dimethyl sulfoxide (DMSO), ammonia (28%), and ethanol (EtOH) were purchased from Tianjin Huihang Chemical Technology Co., Ltd. (Tianjin, China). DL-sulforaphane standard (SFN, purity of 97%), 1,3,5-benzenetricarboxaldehyde (Tb), benzidine (Bd), 1,3,5-tris (4-aminophenyl) benzene (TAPB), and m-phthalaldehyde (MPA) were provided by Shanghai Macklin Biochemical Co., Ltd. (Shanghai, China). Water was purified by a Milli-Q Advantage A10 system (USA). All the reagents were analytical grade at least.

Considering the wide variation in SFN content in different cruciferous vegetables, 10 cruciferous vegetable samples including broccoli, cauliflower, cabbage, Brassica campestris, Chinese cabbage, carrot, red radish, white radish, green turnip, and pak choi (*Brassica chinensis* L.) were purchased from a local supermarket in Beijing, China. All vegetable samples were homogenized by a blender before sample pretreatment.

### 2.2. Instruments and HPLC-MS/MS Conditions

The structures and morphologies of prepared Fe_3_O_4_, COFs, and MB-COFs were observed by transmission electron microscopy (TEM; JEM-200CX, JEOL, Tokyo, Japan) and field emission scanning electron microscopy (SEM; SM-6300, JEOL, Japan). The power X-ray diffraction (XRD) patterns were recorded on a D8 Advance (Bruker AXS GmbH, Karlsruhe, Germany). X-ray photoelectron spectroscopy (XPS, Escalab 250Xi, Thermo Scientific, Waltham, MA, USA) was performed for surface analysis. Fourier-transform infrared (FT-IR, Nicolet 6700, Thermo Scientific) spectra were obtained over the wavenumber range of 400–4000 cm^−1^. The nitrogen adsorption–desorption isotherms were obtained from an ASAP2020 analyzer (Micromeritics Instrument Corp., Norcross, GA, USA) to calculate the Brunauer–Emmett–Teller (BET) surface area and porosity of the MB-COFs. Magnetization curves were measured by a vibrating-sample magnetometer (VSM; BKT-4500, Beijing, China).

Separation and determination of SFN were performed by an HPLC-MS/MS system (LC-30A, MS8050, Shimadzu Corp., Kyoto, Japan) equipped with an electrospray ionization source (ESI). A Kinetex C18 column (50 mm × 3 mm, 2.6 μm, Phenomenex, Torrance, CA, USA) was held at 40 °C with a flow rate of 0.3 mL/min. Gradient elution was applied with water containing 1 mmol/L ammonium acetate (A) and methanol (B) as follows: 0–5 min, 20–100% B; 5–6 min, 100% B; 6–6.1 min, 100–20% B; 6.1–9 min, 20% B. The injector temperature was 4 °C and the injection volume was 1 μL. The MS was operated in positive ion mode with multiple reaction monitoring (MRM) mode. The molecular information, ESI, and MS parameters are listed in Table S1, and the ion chromatogram and mass spectrum of SFN (CAS number: 4478-93-7, Retention time: 2.52 min) in MeOH are shown in Figure S1.

### 2.3. Preparation of Standard Solutions

SFN standard stock solutions (5000 mg/L) were prepared in HPLC-grade methanol and stored at −20 °C for 6 months. Standard working solutions were prepared by serially diluting the stock solutions to obtain the concentrations required for plotting a calibration curve (0.5, 0.2, 0.1, 0.05, 0.02, 0.01, 0.005, and 0.001 mg/L) and an adsorption experiment (0.1–250 mg/L). A matrix-matched standard calibration curve (0.001–0.5 mg/L) was obtained by diluting the standard stock solution with vegetable matrix extracts. All standard solutions were stored at 4 °C in brown glass vials until use.

### 2.4. Preparation of MB-COFs

#### 2.4.1. Preparation of Fe_3_O_4_@PEG

Fe_3_O_4_ nanoparticles were synthesized according to previous literature with some modifications [22]. In brief, FeCl_3_-6H_2_O (1.2 g) and FeSO_4_-7H_2_O (0.7 g) were dissolved in 10 mL of water under ultrasound, respectively, and then filtered into a three-necked flask containing 240 mL of water. After stirring at 80 °C for 30 min, 10 mL of ammonia (28%) was added, and stirring was continued for another 30 min. The suspension was cooled, magnetically separated, and washed several times with deionized water and ethanol, respectively. Afterward, 100 mL of PEG solution (0.5 mmol/L) was added and stirred for 30 min. Finally, the Fe_3_O_4_@PEG was obtained by magnetic separation.

#### 2.4.2. Preparation of Fe_3_O_4_@COFs-1

The COFs-1 were prepared according to previous work with some modifications [23]. The prepared Fe_3_O_4_@PEG was dispersed in 260 mL of DMSO and transferred to a conical flask. Then, 20 mL of DMSO containing 0.3645 g of Tb and 20 mL of DMSO containing 0.6218 g of Bd were added and sonicated for 5 min. HAc (10 mL) as a catalyst was slowly added under magnetic stirring, and the resulting solution was stirred for 1 h under ambient temperature. The obtained Fe_3_O_4_@COFs-1 were collected by magnetic separation and washed twice with deionized water and DMSO alternately, followed by being freeze-dried under a vacuum for 12 h. As a comparison, bare COFs-1 were synthesized in the same way without the addition of Fe_3_O_4_@PEG.

#### 2.4.3. Preparation of Fe_3_O_4_@COF-on-COF

The COFs-2 were prepared following a reported procedure with some modifications [24]. Fe_3_O_4_@COF-on-COF was synthesized with different amounts of ligands that are constitutive of COFs-2 (Table S2). Firstly, the prepared Fe_3_O_4_@COFs-1 (0.5 g) were dispersed in 60 mL of DMSO, and then 60 mL of DMSO containing TAPB and MPA was added to the above solution followed by being sonicated for 5 min. HAc (6 mL) was slowly added dropwise under magnetic stirring at room temperature, and the resulting solution was stirred for 1 h. The final Fe_3_O_4_@COF-on-COF (namely MB-COFs) was collected by magnetic separation, and washed twice with deionized water and DMSO in turns, followed by dried under vacuum for 12 h. As a comparison, bare COFs-2 were synthesized in the same way as MB-COFs without the addition of Fe_3_O_4_@COFs-1.

### 2.5. Sample Preparation

#### 2.5.1. Sample Enzymolysis

The enzymolysis of glucosinolate to SFN from vegetable samples was performed according to previous literature with slight modifications [25]. Five grams of homogenized sample (one gram for broccoli) was hydrolyzed in 20 mL of ultrapure water and incubated in a water bath for 2 h at 45 °C using a thermostatic oscillator (SHA-B, Changzhou Huapuda Teaching Instrument Co., Ltd., Changzhou, China). After cooling to room temperature, the mixture was centrifuged at 8000 r/min for 5 min. Two milliliters of the resulting supernatant was taken for the following MSPE procedure.

#### 2.5.2. MSPE Procedure

Typically, 2 mL of the supernatant was added into a 5 mL centrifuge tube containing 20 mg of MB-COFs. After shaking for 5 min, the MB-COFs with adsorbed SFN were separated magnetically from the solution. Then, SFN was desorbed from the MB-COFs by adding methanol (4 mL) under a vigorous vortex for 30 s. After that, the eluate was collected by magnetic separation and filtered with a 0.22 μm nylon membrane. Depending on the content of SFN in different vegetables, the resulting solution was diluted to the detection concentration within the linear range of LC-MS/MS analysis.

### 2.6. Adsorption Experiment

#### 2.6.1. Optimization of the MSPE Conditions

The MSPE parameters (including adsorption and elution conditions) for SFN were systematically evaluated using the one-factor-at-a-time (OFAT) approach. The initial concentration of SFN was 50 mg/L. For adsorption conditions, the adsorbent dosage (2–50 mg), adsorption time (30 s–90 min), salt concentration (0–10%), pH (2–12), and temperature (25–65 °C) were optimized in detail. The elution conditions were optimized through the desorption solvent (MeOH, EtOH, AC, ACN, and IPA), desorption volume (1–6 mL), and desorption time (30 s–60 min). To investigate the reusability of the adsorbent, the MSPE experiments were repeated by freeze-drying the used adsorbent after washing it several times with MeOH and water. All of the experiments were carried out in three parallels. The adsorption efficiency (E_a_) and desorption efficiency (E_d_) of the adsorbent were calculated according to the following equations [24]:

(1) $$E_{a}\left(\%\right)=\frac{\left(C_{0}-C_{a}\right)}{C_{0}}\times 100\%$$

(2) $$E_{d}\left(\%\right)=\frac{C_{d}\times V_{d}}{\left(C_{0}-C_{a}\right)\times V_{0}}\times 100\%$$

where C_0_, C_a_, and C_d_ (mg/L) represent the concentrations of SFN initially, after adsorption, and after desorption, respectively. V_0_ and V_d_ (L) are the initial solution volume and desorption solvent volume, respectively.

#### 2.6.2. Adsorption Kinetics

To study the adsorption kinetics of MB-COFs toward SFN, 20 mg of adsorbent was added to 2 mL of SFN aqueous solution (200 mg/L), which was shaken with a mixer (SCI-M, SCILOGEX, Rocky Hill, CT, USA) at room temperature for different times (30 s–120 min). Afterward, the mixture was separated by an external magnetic field, and the supernatant was filtered for HPLC-MS/MS analysis after dilution. The adsorption capacity was calculated according to the following equations [26]:

(3) $$Q_{t}=\left(C_{0}-C_{t}\right)\times \frac{V}{m}$$

(4) $$Q_{e}=\left(C_{0}-C_{e}\right)\times \frac{V}{m}$$

where Q_t_ and Q_e_ (mg/g) are the adsorption capacity of SFN at time t and at equilibrium, respectively; C_0_, C_t_, and C_e_ (mg/L) are the SFN concentrations initially, at time t, and at equilibrium, respectively; and V (L) and m (g) are the solution volume and the mass of MB-COFs, respectively.

#### 2.6.3. Adsorption Isotherm

Adsorption isotherm experiments were based on the concentration change of SFN (1, 10, 50, 100, 150, 200, and 250 mg/L) for 30 min at different temperatures (35, 45, 55, and 65 °C). A 20 mg amount of adsorbent was added to 2 mL of aqueous SFN solution with different concentrations, which was shaken with a thermostatic oscillator at different temperatures. Afterward, the mixture was isolated magnetically and the supernatant was filtered and diluted for HPLC-MS/MS analysis.

### 2.7. Method Validation

The method validation was performed according to ICH guidelines [27]. The developed method was validated for SFN in four vegetable matrices (broccoli, cabbage, red radish, and pak choi). A series of validation parameters, including the linearity range, correlation coefficient (R^2^), limit of detection (LOD), limit of quantification (LOQ), accuracy, precision, and matrix effect (ME), were used to evaluate the performance of the proposed method. Linearity was studied by plotting a matrix-matched standard calibration curve against their peak areas. LOD (μg/L) and LOQ (μg/L) were defined as signal-to-noise ratios of 3:1 and 10:1, respectively. Accuracy and precision data were obtained through recoveries and relative standard deviations (RSDs) of the spiked samples at three levels (0.5, 1, and 2 times the SFN content in four vegetables) and each level was conducted in sextuplicate (*n* = 6). ME was evaluated by comparing the slope of the matrix-matched standard calibration curve with that of the standard solvent calibration curve. ME < 1 and >1 indicated signal inhibition and an enhancement effect induced by the matrix, respectively [28].

### 2.8. Statistical Analysis

All experiments were performed at least in triplicate and the experimental data were expressed as mean ± standard deviation (SD). The data were analyzed by a one-way analysis of variance (ANOVA) followed by least significant difference (LSD) and Duncan’s tests using IBM SPSS Statistics 19.0 software to determine the significance level (*p* < 0.05).

## 3. Results and Discussion

### 3.1. Preparation, Optimization, and Characterization of MB-COFs

**Figure 1:**
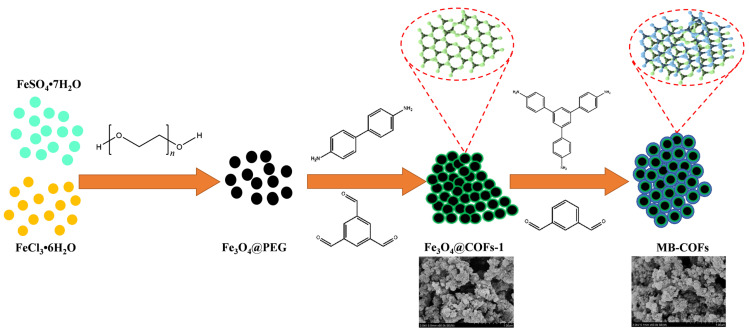
Schematic for formation of MB-COFs.

The preparation procedures of MB-COFs are illustrated in Figure 1. Firstly, Fe_3_O_4_ nanoparticles were prepared by a typical solvothermal reaction with a slight modification. In this step, PEG was used as a polymer stabilizer and modifier to improve the dispersion of Fe_3_O_4_ nanoparticles and reduce their agglomeration. Therefore, after adding PEG, the fabricated Fe_3_O_4_@PEG not only possessed high water dispersion stability but also facilitated the coating of the COFs-1 [29,30]. Then, according to a monomer-mediated in situ growth strategy, two reactive monomers, Tb and Bd, were grown in situ on the Fe_3_O_4_@PEG surface to obtain core–shell Fe_3_O_4_@COFs-1 through the Schiff base reaction in DMSO. Finally, COFs-2 were formed on the surface of Fe_3_O_4_@COFs-1 through the aldehyde ammonium condensation reaction of TAPB and MPA to synthesize magnetic Fe_3_O_4_@COF-on-COF (denoted as MB-COFs). In this process, the COFs-2 were covalently attached to the surface of COFs-1 by means of layer-by-layer assembly at room temperature and pressure, forming a staggered layered-sheet stacking structure with an interlaced pore network [20,21].

**Figure 2:**
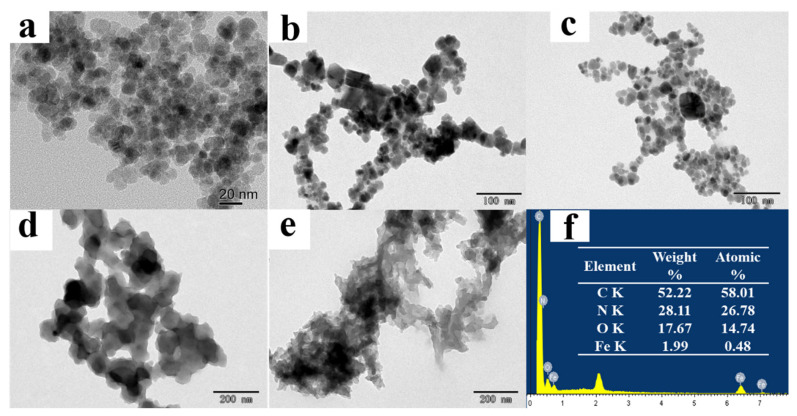
TEM images of Fe_3_O_4_ (**a**), Fe_3_O_4_@COFs-1 (**b**), MB-COFs-4 (**c**), bare COFs-1 (**d**), and bare COFs-2 (**e**); EDS spectra of MB-COFs-4 (**f**).

**Figure 3:**
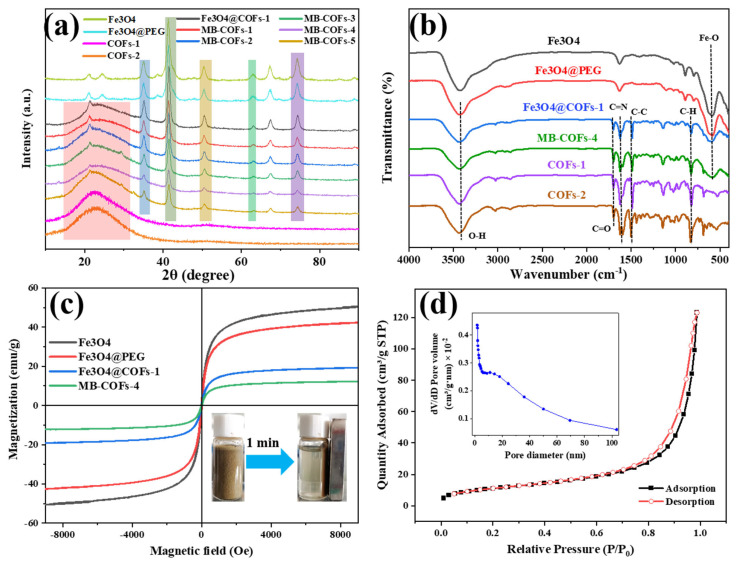
XRD (**a**), FT-IR (**b**), and VSM (**c**) of prepared samples, and N_2_ adsorption–desorption isotherms of MB-COFs-4 (**d**).

In addition, the effect of the amount of COFs-2 on the adsorption properties of the prepared MB-COFs was investigated by controlling the concentration of the ligands of COFs-2 (Table S2). The SEM, EDS (Figure S2), TEM (Figure 2) and XRD (Figure 3) results of MB-COFs were similar for different amounts of COFs-2 obtained by adjusting the concentrations of TAPB and MPA, indicating that the MB-COFs’ structure and crystallinity were not significantly changed during the conditioning process. To obtain the most optimal MB-COFs, the adsorption efficiency of MB-COFs (1–5) for SFN was compared according to the procedure of adsorption experiments. As shown in Table S2, the bare COFs-1 and COFs-2 had the lowest adsorption efficiency (21.15–79.69%) in all SFN concentrations due to the low density of COFs, which caused them to float above the solution instead of being uniformly dispersed in the solution. In contrast, the adsorption efficiency of the Fe_3_O_4_@COFs-1 was increased to 83.06–86.87% thanks to the introduction of Fe_3_O_4_ that endowed the adsorbent with good dispersibility. Furthermore, the adsorption efficiencies of MB-COFs (1–5) increased significantly and reached a plateau with the increased dosage of TAPB and MPA. Especially for MB-COFs-4 and MB-COFs-5, the adsorption efficiency increased by about 18–72% compared to bare COFs. These results provided evidence that MB-COFs had higher adsorption efficiency for SFN than that of monolayer COFs. The adsorption capacity of MB-COFs was dependent on their physicochemical and structural properties, which were further analyzed by various characterization techniques and adsorption experiments as detailed in the following sections. As a result, MB-COFs-4 were selected for the following investigation.

#### 3.1.1. SEM and TEM Analysis

The morphologies of as-synthesized samples were characterized by using TEM and SEM. TEM images of Fe_3_O_4_ demonstrated that spherical Fe_3_O_4_ had a smooth surface with good dispersion and a diameter of about 20 nm (Figure 2a). Figure 2b,c show the TEM images of Fe_3_O_4_@COFs-1 and MB-COFs-4, respectively. It can be seen that Fe_3_O_4_@COFs-1 and MB-COFs-4 presented a spherical cluster-like structure with rough surfaces and particle sizes similar to Fe_3_O_4_@PEG after being successively coated by COFs-1 and COFs-2. In comparison, bare COFs-1 and COFs-2 exhibited larger-size amorphous stacked structures (Figure 2d,e). This obvious difference indicated that the COF layer was successfully synthesized on the surface of Fe_3_O_4_@PEG. The SEM images of Fe_3_O_4_@COFs-1 and MB-COFs were similar for different amounts of COFs-2, which showed a particulate cluster structure with abundant pores (Figure S2). To further explore the elemental composition changes of MB-COFs, the EDS spectra of corresponding SEM images were analyzed and are presented in Figure S2. The original Fe_3_O_4_@PEG had a high content of Fe and O elements, while the presence of the C element was attributed to the introduction of PEG. Compared with the spectra of Fe_3_O_4_, the amount of C and N increased sharply in the Fe_3_O_4_@COFs-1 due to the coated COFs-1. After coating of COFs-2 on the surface of Fe_3_O_4_@COFs-1, the C and N further increased, and the final MB-COFs-4 were composed of C (52.22%), N (28.11%), O (17.67%), and Fe (1.99%) (Figure 2f).

#### 3.1.2. XRD Analysis

The crystalline structures of Fe_3_O_4_, Fe_3_O_4_@PEG, COFs-1, COFs-2, Fe_3_O_4_@COFs-1, and MB-COFs (1–5) were characterized by XRD analysis, and the results are shown in Figure 3a. The diffraction peaks of the MB-COFs (1–5) appearing at 35.2°, 43.1°, 53.2°, 62.9°, and 74.4° were ascribed to the (311), (400), (422), (440), and (533) crystallographic planes of Fe_3_O_4_, respectively, demonstrating that the MB-COFs had good crystallinity and relatively stable crystallographic structures [31,32]. Furthermore, the broad diffraction peak between 15° and 35° showed the presence of the amorphous structure of the COF shell [22]. These results indicated that the MB-COFs were successfully synthesized.

#### 3.1.3. FT-IR Analysis

FT-IR spectra were obtained to determine the functional groups of Fe_3_O_4_, Fe_3_O_4_@PEG, Fe_3_O_4_@COFs-1, MB-COFs-4, COFs-1, and COFs-2. As shown in Figure 3b, the typical absorption peak at 587 cm^−1^ was caused by the stretching vibration of Fe-O, and the absorption bands at 3431 and 1391 cm^−1^ indicated the existence of carboxyl groups. The featured peak of 1622 cm^−1^ was due to the stretching vibration of the C=N bond, suggesting the successful formation of COFs. The signals near 1594 and 1500 cm^−1^ originated from the stretching vibration of the C–C bond in the benzene ring skeleton. The peak near 826 cm^−1^ was attributed to the C–H bending vibration on the benzene ring. The peaks at 1140, 1284, 1697, and 2861 cm^−1^ were ascribed to C-C, C–N, C=O, and C–H stretching vibrations in the COFs, respectively. The FT-IR data were consistent with previous findings [22], further confirming the successful synthesis of COF shells coated on the surface of MB-COFs-4 by the Schiff base reactions.

#### 3.1.4. VSM Analysis

The magnetic properties were investigated using the magnetization hysteresis loop recorded by VSM, as shown in Figure 3c. Clearly, the saturation magnetization decreased with the increase in COF coating on the Fe_3_O_4_ surface, which was 51.3, 43.0, 19.53, and 12.3 emu/g for Fe_3_O_4_, Fe_3_O_4_@PEG, Fe_3_O_4_@COFs-1, and MB-COFs-4, respectively. However, hysteresis, coercivity, or remanence was not found in the magnetization process for all samples, and both magnetic hysteresis loops of the samples had S-shaped curves, showing almost typical superparamagnetic properties. Meanwhile, MB-COFs-4 could be rapidly separated from the uniform dispersion in water in less than 1 min by an external magnet, demonstrating that the prepared adsorbent had sufficient magnetic response performance that made it feasible in the MSPE application.

#### 3.1.5. N_2_ Adsorption–Desorption Isotherms

The porosity of the MB-COFs-4 was confirmed by N_2_ adsorption–desorption isotherms at 77 K and the results are shown in Figure 3d. It could be seen that MB-COFs-4 followed typical type IV isotherms with the obvious hysteresis loop, demonstrating the existence of mesopores. The pore size distribution curve shown in the inset of Figure 3d confirmed that this material had a richer mesoporous structure. Furthermore, the BET and BJH methods were used to determine the specific surface area and pore volume of the samples (Table S3). The surface area, pore volume, and size of Fe_3_O_4_@COFs-1 were 61.7 m^2^/g, 0.27 cm^3^/g, and 15.2 nm, respectively. These values were slightly decreased compared to COFs-1 due to the addition of Fe_3_O_4_. This observation also occurred in other reported Fe_3_O_4_@COFs [24]. Nevertheless, the introduction of Fe_3_O_4_ not only did not affect the excellent physicochemical characteristics of COFs but also imparted superior magnetism to COFs. Interestingly, the coating of COFs-2 on Fe_3_O_4_@COFs-1 reduced the specific surface area and pore volume of MB-COFs-4 to a larger extent, causing the final value to drop to 40.5 m^2^/g and 0.18 cm^3^/g, respectively. This may be attributed to the fact that the attached TAPB and MPA molecules occupied some space in the pores, thus decreasing the pore volume and surface area. These results indicated that the porous structure of MB-COFs was beneficial for enhancing the mass transfer and adsorption of SFN molecules.

#### 3.1.6. XPS Analysis

**Figure 4:**
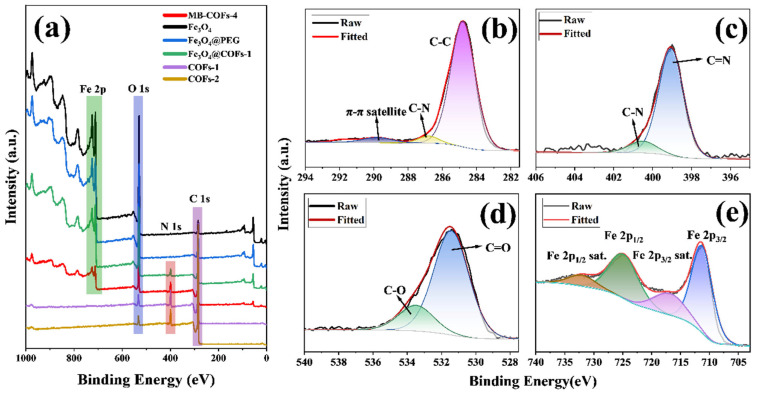
XPS survey of prepared samples (**a**), high-resolution XPS of C 1s (**b**), N 1s (**c**), O 1s (**d**), and Fe 2p regions (**e**) of MB-COFs-4.

To obtain further information about the surface chemical composition and valence states of elements, the XPS spectra of the MB-COFs-4 and other samples were displayed in Figure 4. The wide scan spectra (Figure 4a) of the MB-COFs-4 displayed four strong peaks at binding energies of 712.3, 531.4, 399.0, and 284.8 eV, which belonged to Fe 2p, O 1s, N 1s, and C 1s, respectively. The C 1s spectrum (Figure 4b) can be divided into three peaks, which are assigned to the π-π satellite (289.6 eV), C-N (286.7 eV), and C-C (284.8 eV), respectively [33]. In the N 1s spectrum (Figure 4c), the peaks located at 400.5 and 399.0 eV can be attributed to the C-N and C=N, respectively [17]. Furthermore, the O 1s spectrum (Figure 4d) was centered at 533.5 and 531.4 eV, indicating the presence of C–O and C=O, respectively. In addition, the peaks at 724.8 and 711.3 eV from the deconvolution can be assigned to the Fe in Fe 2p_1/2_ and Fe 2p_3/2_, respectively, and the peaks at 732.0 and 716.8 eV belong to the satellite peaks of Fe [18]. These results indicated that the construction of MB-COFs-4 was successful.

### 3.2. Optimization of MSPE Procedures

#### 3.2.1. Adsorbent Amount

**Figure 5:**
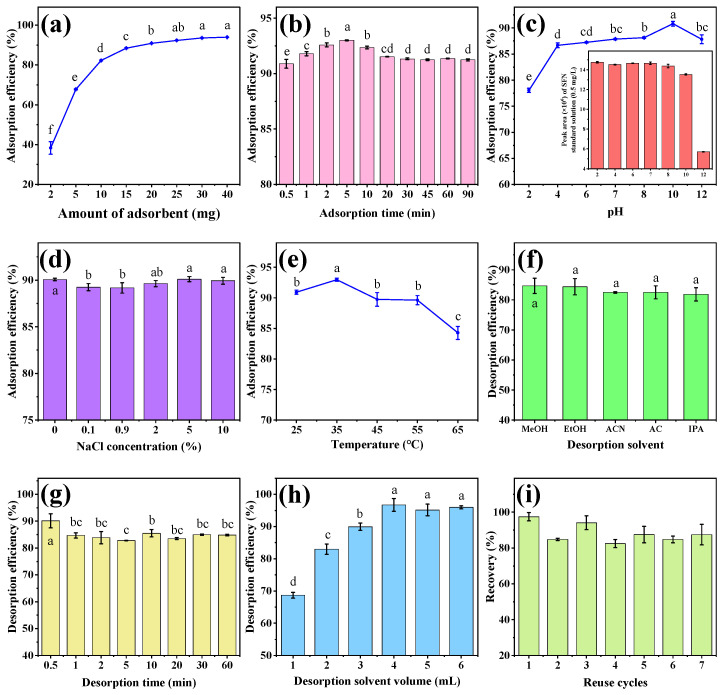
The effect of adsorbent amount (**a**), adsorption time (**b**), pH (**c**), ionic strength (**d**), temperature (**e**), desorption solvent (**f**), desorption time (**g**), and desorption solvent volume (**h**) on adsorption efficiency of MB-COFs-4; reusability of MB-COFs-4 (**i**). Different letters indicate that the difference is significant at the *p* < 0.05 level.

In the MSPE procedure, the amount of adsorbent affects the number of adsorption sites and thus the extraction efficiency of the method. Therefore, different amounts of MB-COFs-4 (2, 5, 10, 15, 20, 25, 30, and 40 mg) were evaluated for SFN extraction to achieve high recovery with an extraction time of 30 min and a sample volume of 2 mL (50 mg/L). As shown in Figure 5a, the adsorption efficiency of SFN increased significantly from 2 to 20 mg (38.3% to 90.8%) and remained almost constant (less than 94%) with a further increase in the amount of MB-COFs-4. This was due to the increased surface area and adsorption sites of MB-COFs-4. Thus, 20 mg of MB-COFs-4 was used in this work.

#### 3.2.2. Adsorption Time

The adsorption time not only influences the adsorption efficiency but also decides the total analysis time. The aim of the MSPE procedure is to achieve adsorption equilibrium between adsorbents and analytes in the shortest possible time. In this work, adsorption times ranging from 30 s to 90 min were studied to gain good adsorption efficiency with 20 mg of MB-COFs-4. As shown in Figure 5b, the adsorption efficiency was at a high level (above 90%) throughout time, indicating that the MB-COFs-4 adsorbent had an ultra-fast adsorption equilibrium time owing to the fast mass transfer and binding kinetics of SFN on MB-COFs-4, which significantly reduced the whole analysis time. Considering the maximum adsorption efficiency, the adsorption time was set to 5 min for further experiments.

#### 3.2.3. pH

Due to the existence of organic acids or alkaloids in vegetables, the pH of the extract affects not only the surface charge and ionization degree of the adsorbent but also the solubility, stability, and form of analytes, thus altering the adsorption efficiency and specificity between the adsorbent and target compound. Herein, the effect of the pH on the adsorption efficiency was investigated in the range of 2–12 adjusted by a HCl or NaOH solution. As shown in Figure 5c, the adsorption efficiency of SFN increased significantly in the pH range of 2–4 and then plateaued with increasing pH (*p* < 0.05). The results exhibited that the MB-COFs-4 had promising adsorption performance over a wide pH range (4–12), which can be applied to the efficient adsorption of SFN under different pH conditions. However, SFN was more stable under acidic and neutral conditions, while it was susceptible to degradation under alkaline conditions with pH greater than 8 (inset of Figure 5c), which was consistent with previous reports [34,35]. Therefore, the optimized pH range of 4–8 indicated that the adjustment of the pH of the sample solution could be avoided. Considering the practical applications, pH 7 was selected for further experiments.

#### 3.2.4. Ionic Strength

The presence of salt ions can increase the viscosity of the aqueous solution and may compete with the target compound for adsorption sites of the adsorbent, thus reducing the adsorption efficiency [36]. The impact of ionic strength on the adsorption efficiency was examined by preparing SFN solutions with different NaCl concentrations (0–10%, *w*/*v*). Figure 5d shows that the adsorption efficiency has no obvious change for SFN, revealing that MB-COFs-4 had good resistance to salt ion interference. Therefore, the effect of ionic strength was no longer considered in the next experiments.

#### 3.2.5. Temperature

Appropriate temperature can promote better dispersion of MB-COFs-4 into the aqueous phase and also accelerate the adsorption of SFN on its surface, thus improving the adsorption efficiency. Herein, the effect of temperature on the adsorption efficiency was investigated in the range of 25–65 °C in a water bath using a thermostatic oscillator. As shown in Figure 5e, the adsorption efficiency increased and then decreased in the ranges of 25–35 °C and 35–65 °C, respectively. This is due to the fact that an appropriate increase in temperature accelerates the diffusive rate of SFN caused by Brownian motion. Nevertheless, too-high temperatures cause enhanced diffusion and dissociation of SFN from the surface of MB-COFs-4, thus resulting in a slight decrease in adsorption efficiency [37]. Therefore, the MSPE operation was performed at room temperature (25–35 °C) in this work.

#### 3.2.6. Desorption Solvent

A suitable desorption solvent enables fast and efficient desorption of SFN from the adsorbent. In this work, five solvents with different polarities including MeOH, EtOH, ACN, AC, and IPA (2 mL of each solvent and 30 min of desorption time) were compared to obtain the optimal desorption efficiency. As presented in Figure 5f, all five solvents eluted SFN well without significant differences (*p* > 0.05), and the desorption efficiency was 81.8–84.7%, with the highest being MeOH. Hence, MeOH was selected as the desorption solvent in the following experiments.

#### 3.2.7. Desorption Time

Desorption time was optimized in the range of 30 s–60 min (Figure 5g). The desorption efficiency for SFN fluctuated in a small range with increasing time due to dynamic desorption equilibrium, with the highest value (90.1%) obtained at 30 s. This result showed that SFN could not only be rapidly adsorbed by MB-COFs-4 but also rapidly desorbed, which greatly improved the efficiency of sample analysis. Thus, the desorption time was chosen as 30 s.

#### 3.2.8. Desorption Solvent Volume

In order to achieve the best desorption efficiency, the effect of MeOH volume (1–6 mL) was optimized. As illustrated in Figure 5h, the desorption efficiency of SFN remarkably went up from 68.7 to 96.7% as the volume increased from 1 to 4 mL (*p* < 0.05), followed by a slight decrease with further addition of volume (*p* > 0.05). Thus, the MeOH volume was fixed at 4 mL for the subsequent experiments.

#### 3.2.9. Reusability

From a practical and economic point of view, the reusable stability and regenerative capacity of adsorbents are critical to be considered. Hence, based on the above optimal conditions, repeated adsorption–desorption cycle experiments were performed and evaluated with the recovery. As shown in Figure 5i, the recovery displayed a slight reduction after the first usage but fluctuated at a high level of 82.5–97.5% over seven cycles. The results exhibited that the MB-COFs-4 absorbent could be reused at least seven times without obvious loss of recovery, revealing the good stability and reusability of the MB-COFs-4.

### 3.3. Adsorption Kinetics

To explore the kinetic behavior between MB-COFs and SFN, several kinetic models, including the pseudo-first-order, pseudo-second-order, Ritchie-second-order, Webber–Morris’s intra-particle diffusion, liquid-film diffusion, Elovich, Frusawa and Smith (F&S), Mathews and Weber (M&W), and Boyd’s models were used to fit experimental data [38,39,40,41,42,43,44]. These models are currently commonly used to analyze the adsorption kinetics of organic compounds by an adsorbent and can reveal the adsorption mechanism from different aspects. The equations of these models are detailed in the Supplementary Materials.

**Figure 6:**
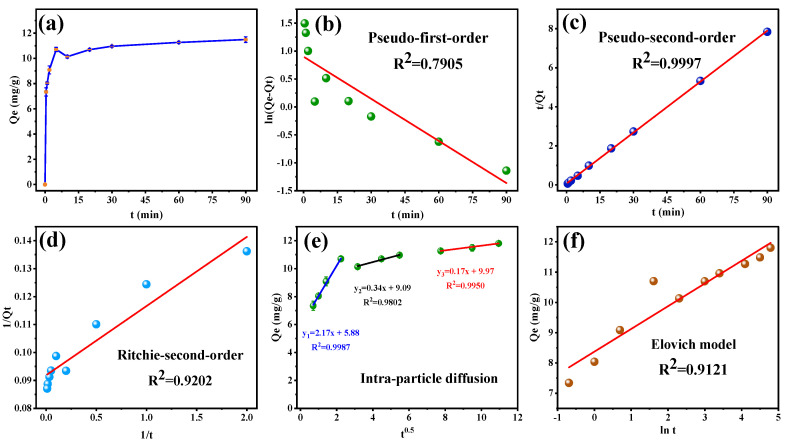
Adsorption amount of SFN at different times (**a**) and the corresponding kinetic models of pseudo-first-order (**b**), pseudo-second-order (**c**), Ritchie-second-order (**d**), intra-particle diffusion (**e**), and Elovich (**f**).

Figure 6a displays the amount of SFN adsorbed at different times (Q_t_). The adsorption amount of SFN raised quickly to more than 90% of the equilibrium adsorption at 5 min, followed by a slow change. The fitting curves of pseudo-first-order, pseudo-second-order, and Ritchie-second-order kinetic models of MB-COFs-4 toward SFN are shown in Figure 6b–d, respectively, and the relevant kinetic parameters are listed in Table S4. It was clearly observed that a higher R^2^ (0.9997) of pseudo-second-order was obtained than those of pseudo-first-order (0.7905) and Ritchie-second-order (0.9202). Moreover, the experimental value of Q_e,exp_ (11.804 mg/g) was nearer to the calculated value (11.501 mg/g) obtained by the pseudo-second-order model. In addition, as shown in Figure 6e and Table S4, the R^2^ value was higher for diffusion in the intra-particle model (0.9802–0.9987) than the liquid-film model (0.7905), thereby implying the intra-particle diffusion might have been the main limitation that affected the adsorption of SFN by MB-COFs-4. The intra-particle diffusion model showed that the adsorption process was divided into three stages, involving surface adsorption, internal diffusion, and adsorption equilibrium, suggesting that the adsorption kinetic was constrained by a multistep mechanism. The first sharp line suggested a rapid adsorption stage owing to the rich adsorption sites and the macropore or large inner space of MB-COFs-4. The second line represented the relatively slow micropore or intra-particle diffusion. The third linearity was very flat and the diffusion control may have turned into a reaction control process due to fewer available binding sites and slower adsorption rates [45]. Furthermore, none of the three lines passed through the origin (C≠0), suggesting that intra-particle diffusion was not the only restricting step in adsorption. Thus, it could be concluded that the adsorption mechanism was complicated and external, and intra-particle diffusion contributed to the actual adsorption process. The Elovich model assumes that the activation energy increases with prolonged adsorption time and that the surface of the adsorbent has a heterogeneous surface [44]. As depicted in Figure 6f, the good fit (R^2^ = 0.9121) of the Elovich model suggested that the increase in activation energy was due to the heterogeneous surface of the adsorbent, confirming that the kinetics of SFN adsorption on MB-COFs-4 followed a surface interaction mechanism by active sites. In addition, the high value of α (713.46 mg⋅g^−1^⋅min^−1^) in the Elovich equation indicated a very high initial adsorption rate and a fast and effective coverage of the adsorbent surface by adsorption. The low value of β (1.333 mg/g) verified that desorption was a non-preferential process that kept the adsorption of SFN at active sites. Hence, the parameters of the Elovich equation showed that MB-COFs-4 have many active sites distributed on their surfaces [46]. F&S, M&W, and Boyd’s models were applied to determine the external mass transfer in the adsorption process [42]. A comparison of correlation coefficients (Table S4) showed that Boyd’s external diffusion equation surpassed the F&S and M&W models. However, none of the linear fits of the three models intersected the origin and the experimental data points were scattered, suggesting that the adsorption of SFN by MB-COFs-4 was mainly governed by external mass transport, where diffusion in the film was the rate-limiting step [38]. Also, the β_1_S values obtained by the F&S (0.00264 s^−1^) and M&W (0.00265 s^−1^) model equations were consistent, which indicated that the velocity of SFN transport from the liquid phase to the surface of MB-COFs-4 was sufficiently rapid [42]. Therefore, the pseudo-second-order, Ritchie-second-order, intra-particle diffusion, and Elovich kinetic models best fitted the experimental data, indicating that the adsorption was a combination of physisorption and chemisorption, with the latter predominating, and the intra-particle diffusion might be the rate-limiting step.

### 3.4. Adsorption Isotherm

**Figure 7:**
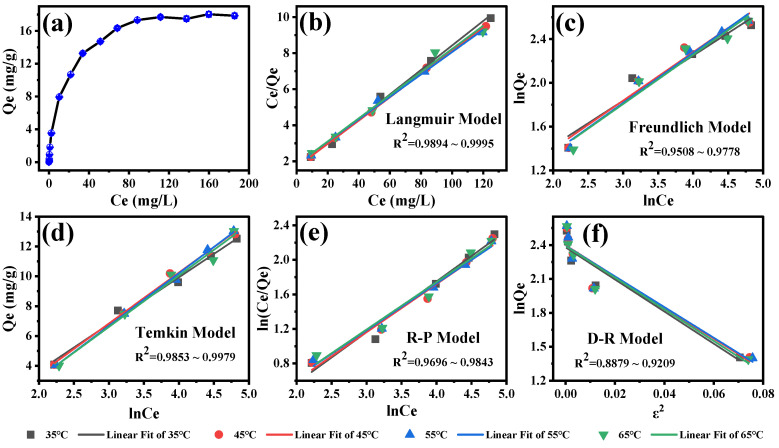
Adsorption amount of SFN at different initial concentrations (**a**) and the corresponding isotherm models of Langmuir (**b**), Freundlich (**c**), Temkin (**d**), Redlich–Peterson (**e**), and Dubinin–Rabushkevich (**f**).

To further investigate the adsorption isotherm of SFN by MB-COFs-4, Langmuir, Freundlich, Dubinin–Rabushkevich (D-R), Temkin, and Redlich–Peterson (R-P) models were used to fit the experimental data [43,46,47,48,49,50]. These models are currently commonly used to analyze the adsorption isotherm of organic compounds by the adsorbent and can reveal the adsorption mechanism from different aspects. The linear equations of these models are detailed in the Supplementary Materials. Figure 7a shows the adsorption amount of SFN at different initial concentrations. With the increase in the initial concentration of SFN from 0.1 to 250 mg/L, the adsorption capacity first increased quickly and finally achieved adsorption equilibrium, with the maximal adsorption capacity of 18.0 mg/g. This is due to the fact that the number of collisions between the SFN molecule and the adsorbent increases with rising initial SFN concentration, which leads to an improvement in the adsorption capacity. SFN adsorption isotherms and equilibrium parameters that were calculated by fitting Langmuir, Freundlich, D-R, Temkin, and R-P models at 35–65 °C are presented in Figure 7b–f and Table S5. The results showed that the Langmuir isotherm model has the highest R^2^ values (0.9894–0.9996) compared to the other models, indicating that the Langmuir isotherm model was more suitable for describing the adsorption process of MB-COFs-4 on SFN. The good fit found for the Langmuir model confirmed that the homogenous surface of the MB-COFs-4 and the adsorption process correspond to monolayer coverage [51]. The R_L_ values shown in Table S5 indicate a favorable adsorption process for all temperatures. These results corroborate the good fit for the pseudo-second-order and Elovich kinetic parameters presented above. In addition, the 1/n values of less than 1 in the Freundlich model were observed for all temperatures, indicating favorable adsorption. The results of the Temkin model showed a satisfactory fit for all temperatures (R^2^ ≥ 0.9775), confirming that the SFN adsorption was characterized by a uniform distribution of binding energy, which corroborated the results obtained by the Langmuir model and pseudo-second-order, Ritchie, and Elovich models [47]. The fit of the R-P model was also considered good (R^2^ of 0.9696–0.9898) and similar to that obtained in the Langmuir model. Nevertheless, the D-R isotherm plot did not fit the experimental data well, with the lowest R^2^ values (0.8879–0.9254) compared to the above four isotherm models. Thus, the adsorption isotherm data were highly consistent with the Langmuir, Temkin, and Redlich–Peterson models, demonstrating that the SFN adsorption was monolayer-favorable adsorption, which was consistent with the conclusion based on the good fit to the kinetic model [49].

### 3.5. Adsorption Thermodynamics

To further account for the adsorption thermodynamic behavior of MB-COFs-4 for SFN, the adsorption experiments were also carried out at 298, 308, and 318 K for three initial concentrations of SFN (1, 10, 100 mg/L), respectively, and thermodynamic parameters including changes in the Gibbs free energy (ΔG, kJ/mol), adsorption enthalpy (ΔH, kJ/mol), and adsorption entropy (ΔS, J mol^−1^ K^−1^) were calculated by the following equations [41,45].

(5) $$K_{e}=\frac{Q_{e}}{C_{e}}$$

(6) $$K_{d}={\;K}_{e}\;\times \rho_{w}$$

(7) $$\Delta G={-RTlnK}_{d}$$

(8) ΔG=ΔH−TΔS

(9) $${lnK}_{d}=\frac{\Delta S}{R}-\frac{\Delta H}{RT}$$

where C_e_ (mg/L), Q_e_ (mg/g), *R* (8.314×10^−3^ kJ mol^−1^ K^−1^), and *T* (K) represent the same meaning as those in the above equations. *K_e_* (L/g) is the ratio of the adsorption amount at equilibrium, *K_d_* is a dimensionless adsorption equilibrium constant, and *ρ_w_* is the density of the water (1000 g/L).

The values of ΔS and ΔH were calculated from the intercept and slope of Vant Hoff’s thermodynamic plot of lnK_d_ versus 1/T (Figure S3). The values of K_d_, ΔG, ΔH, and ΔS are summarized in Table 1. The negative values of ΔG under all experimental conditions confirmed that the SFN adsorption on MB-COFs-4 was spontaneous. Furthermore, the ΔG values gradually increased with increasing temperature, suggesting that adsorption is a spontaneous exothermic process, and higher temperature may be unfavorable for the adsorption process [52]. ΔH < 0 again indicated that the adsorption was exothermic in nature, in accordance with the adsorption capacity decreasing at higher temperatures. In addition, ΔH values between 2 and 21 kJ/mol imply physisorption, while values between 80 and 200 kJ/mol mean chemisorption [53]. For low initial concentrations of SFN (1 and 10 mg/L), 32.65–35.91 kJ/mol was between physical and chemical adsorption, demonstrating that the adsorption of SFN on MB-COFs-4 was physicochemical adsorption, rather than single physical or chemisorption. Moreover, it could be deduced that the main forces were hydrogen bonding and intermolecular van der Waals forces since ΔH < 0 and ΔS < 0 [54]. These results agree with results concluded from adsorption isotherms and kinetics. Notably, for a high concentration (100 mg/L), the ΔH and ΔS values were significantly different from those at low concentrations, which may be due to the fact that high concentrations of SFN occupied all the adsorption sites of MB-COFs-4. In this case, the randomness of the solid–liquid interface increased during the adsorption process, resulting in a weakening of the adsorption capacity of SFN. Instead, the desorption process is spontaneous, and the internal structure of MB-COFs-4 may have changed significantly [45].

### 3.6. Adsorption Mechanism

To better understand the adsorption behavior of MB-COFs-4 to SFN, it is necessary to investigate the adsorption mechanism. According to the characterization and the kinetic and isothermal adsorption models of MB-COFs-4, the adsorption mechanism between the composite and SFN may have hydrogen bonding, π-π stacking, and electrostatic interactions. The partially protonated nitrogen and macrocyclic cavity of the aromatic ring of MB-COFs-4 give them a unique porous structure framework, a large conjugated system, and multiple recognition sites [22]. During the synthesis of COF materials, it is reported that π-π stacking occurs between benzene rings, and the π-conjugated system rich in nitrogen structures and electrons can provide effective binding sites for adsorption [55]. During the adsorption process of SFN, the amino group and the aldehyde group react to form acetal amine and hydrogen bonds (C-H···O-H or C-H···O=C), and the strength of the adsorption capacity is also related to the presence of hydrogen bonds [56]. Other possible interactions between MB-COFs and SFN include van der Waals, N=H⋅⋅⋅π, and C-S⋅⋅⋅π interactions.

### 3.7. Method Validation and Comparison

Under the optimal MSPE conditions, an MSPE-HPLC-MS/MS method was proposed for the determination of SFN in vegetable samples. As shown in Table 2, the linear ranges were 0.001–0.5 mg/L with R^2^ values ≥ 0.9923. The LODs and LOQs were in the ranges of 0.052–0.092 μg/L and 0.18–0.31 μg/L, respectively. Therefore, the developed method was applicable to the analysis of SFN in vegetables. In addition, MEs were <1 (0.52–0.83) for all vegetable matrices, indicating that there was a matrix suppression effect for SFN. More importantly, it can be clearly seen that the matrix suppression effect was significantly reduced to a negligible level (0.82–0.97) after MSPE purification, demonstrating that matrix interference can be significantly reduced using the MB-COFs-4 adsorbent. The vegetable samples spiked with three levels of SFN were analyzed (Table 3). The recoveries were in the range 82.2–96.2% with RSD ≤ 7.9%, indicating that the developed method is reliable and can be used to directly extract and determine SFN in vegetable samples.

The performance of the present method was compared with previous reported methods for the determination of SFN in vegetables (Table 4). The results show that the analytical properties of the method are comparable or even better than other methods. The LOD of the developed method is much lower than these reported methods. Also, the linear range of this method is wider than other methods. Remarkably, the extraction time in this work is about 8 min, which is considerably less than all the reported extraction times thanks to the rapid adsorption–desorption and magnetic separation of MB-COFs. Meanwhile, the proposed method consumes less organic solvent than the reported methods from the perspective of green chemistry. In conclusion, the MSPE-HPLC-MS/MS method can reduce the consumption of organic solvents, save time and cost, and can be used for the rapid and sensitive determination of SFN in complex samples.

### 3.8. Real Sample Analysis

To evaluate the practical applicability of the proposed method, the determination of SFN in ten cruciferous vegetables was performed (Figure S4 and Table S6). SFN content in fresh cruciferous vegetables was highly variable, ranging from 0.01 to 36.78 mg/kg FW. Chromatographic analysis results showed the impurity peaks disappeared or decreased significantly after purification by MSPE. The results showed that the developed method was equally applicable to other cruciferous vegetables with widely varying SFN contents, indicating its good applicability.

## 4. Conclusions

In the present work, MB-COFs were successfully fabricated through a monomer-mediated in situ growth strategy and a layer-by-layer assembly method and applied as the adsorbent of MSPE coupled with HPLC-MS/MS for the rapid adsorption and quantification of SFN in cruciferous vegetables. Techniques including SEM, EDS, TEM, FT-IR, XRD, XPS, and VSM were utilized to characterize the as-prepared MB-COFs, which showed a spherical cluster-like structure with rough surfaces and abundant mesopores, adequate magnetism, and good stability and reusability. In addition, benefiting from the enhanced interaction of dual-layer COFs, the adsorption efficiency of MB-COFs for SFN increased by about 18–72% compared to bare COFs. The MB-COFs displayed a maximum adsorption capacity of 18.0 mg/g and a short adsorption–desorption time for SFN. The pseudo-second-order, Ritchie-second-order, intra-particle diffusion, and Elovich kinetic models and Langmuir, Temkin, and Redlich–Peterson isotherm models best fitted the experimental data, indicating that the adsorption was a combination of physisorption (via van der Waals interaction, π-π stacking, electrostatic interactions, N=H⋅⋅⋅π, C-S⋅⋅⋅π interactions) and chemisorption (via hydrogen bonding), and the intra-particle diffusion might be the rate-limiting step. By using MB-COFs as adsorbents, the proposed method displayed a short analysis time, extremely low organic solvent consumption, wide linearity, excellent sensitivity, satisfactory accuracy and precision, and negligible ME, and it can be employed as a routine method for the extraction and determination of SFN in cruciferous vegetables. This work not only reveals the good application potential of MB-COFs in sample preparation but also provides a more rapid and sensitive method with less organic solvent for the detection of SFN in large-scale vegetable samples in practical applications.

## Figures and Tables

**Table 1:** Analysis of adsorption thermodynamics of MB-COFs-4 on SFN with different initial concentrations.

C_0_ (mg/L)	*T* (K)						ΔH (kJ/mol)	ΔS (J mol^−1^ K^−1^)
	298 K		308 K		318 K			
	*K*_*d*_	ΔG (kJ/mol)	*K*_*d*_	ΔG (kJ/mol)	*K*_*d*_	ΔG (kJ/mol)		
1	2769.1	−19.64	2024.1	−19.49	1109.2	−18.54	−35.91	−54.16
10	2003.3	−18.84	1320.2	−18.40	874.4	−17.91	−32.65	−46.33
100	393.3	−14.80	338.6	−14.92	302.9	−15.11	−10.31	15.05

**Table 2:** Linearity, correlation coefficients (R^2^), LODs, LOQs, and MEs for SFN in vegetables.

Matrix	Linear Range (mg/L)	Regression Equation	R^2^	LOD (μg/L)	LOQ (μg/L)	ME	
						Before MSPE	After MSPE
MeOH	0.001–0.5	Y = 47,437,718x + 333,257	0.9976	0.035	0.12	—	
Pak choi	0.005–0.5	Y = 41,538,858x + 187,6076	0.9951	0.092	0.31	0.74	0.88
Broccoli	0.001–0.5	Y = 45,794,584x + 231,5048	0.9923	0.057	0.19	0.83	0.97
Cabbage	0.001–0.5	Y = 42,573,695x + 3,863,071	0.9962	0.052	0.18	0.71	0.90
Radish	0.001–0.5	Y = 39,017,313x + 6,545,277	0.9985	0.073	0.24	0.52	0.82

**Table 3:** Recoveries and relative standard deviations (RSDs) of SFN in vegetables (*n* = 6).

Matrix	Original (mg kg^−1^)	Spiked (mg kg^−1^)	Recovery/%	RSD/%
Pak choi	0.14	0.1	90.0	4.5
		0.2	91.6	4.5
		0.4	93.6	1.8
Broccoli	45.62	20	92.6	3.4
		40	96.2	3.5
		80	94.2	3.9
Cabbage	3.28	2.5	91.2	4.6
		5	82.2	3.8
		10	88.1	3.1
Radish	7.38	5	92.5	7.9
		10	89.3	3.6
		20	95.1	2.7

**Table 4:** Comparison of the proposed method with other analytical techniques for the determination of SFN in vegetables.

Sample	Methods	Extraction Time or Frequency	Solvents	Linear Range (mg/L)	Recovery (%)	RSD (%)	LOD (μg/L)	LOQ (μg/L)	Ref.
Pak choi, Broccoli, Cabbage, Radish	MSPE-HPLC-MS/MS	7.5 min	4 mL MeOH	0.001–0.5	82.2–96.2	1.8–7.9	0.035–0.092	0.18–0.31	This work
*Raphanus sativus* L. var. *caudatus* Alef	LLE-HPLC-DAD	3 times	DCM	5–40	96.8	0.51	360	1080	[57]
Broccoli	SPE-HPLC-UV	3 times	24 mL DCM, 4 mL MeOH and 4 mL ethyl acetate	0.05–200	90.8–96.4	<3.6	20	/	[58]
Broccoli and Cabbage	LLE-HPLC-DAD	2 times	50 mL DCM	2.5–17.5	95.6	1.2	/	/	[59]
Broccoli	LLE-UPLC–MS/MS	3 times	50 mL DCM	1–10	/	/	77	235	[25]
Broccoli	SPE-HPLC-UV	3 times	60 mL DCM	0.3–250	98.4	1.38	/	/	[60]
Brassicaceae vegetables	DLLME- HPLC-DAD	/	1 mL ACN and 0.7 mL chloroform	5–100	80–110	<15	100–220	300–740	[4]
Broccoli	HPLC-UV	3 times	100 mL DCM	50–400	>96	/	/	/	[61]
Broccoli by-products	SPE-HPLC-UV	>3 h	20 mL DCM	4–80	97.5–98.1	3–4	580	/	[62]
Broccoli and red cabbage	LLE-HPLC-DAD	/	800 mL DCM	0.09–0.36	95.1	3.8	29.7	90	[63]
Broccoli	LLE-HPLC-DAD	/	25 mL methyl t-butyl ether	0.6–200	92–102	4–5	200	600	[64]

## Data Availability

Data is contained within the article and Supplementary Materials.

## Supplementary Materials

The following supporting information can be downloaded at: https://www.mdpi.com/article/10.3390/foods13030409/s1, Figure S1: The ion chromatogram (a) and mass spectrum (b) of SFN in MeOH (0.01 mg/L), Figure S2: SEM (a–c, g–i) and EDS (d–f, j–l) results of Fe3O4@COFs-1 and MB-COFs (1–5), respectively, Figure S3: Vant Hoff’s thermodynamic plot for adsorption of SFN, Figure S4: Extracted ion chromatograms of SFN in real cruciferous vegetable samples before and after MSPE. Pakchoi and carrot (a); red radish and Chinese cabbage (b); cabbage, broccoli and green turnip (c); and white radish, Brassica campestris, and cauliflower (d), Table S1: The molecular information, ESI, and MS/MS parameters of SFN, Table S2: MB-COFs with different amounts of ligands and their adsorption efficiency for SFN with different concentrations (mg/L), Table S3: Surface area, pore volume, and size of prepared samples, Table S4: Parameters of kinetic models for SFN adsorption by MB-COFs-4, Table S5: SFN adsorption equilibrium parameters in MB-COFs-4 at different temperatures, Table S6: SFN content in MB-COFs-4 in fresh cruciferous vegetables.

## References

[1] Sikorska-Zimny K., Beneduce L. (2021). The glucosinolates and their bioactive derivatives in Brassica: A review on classification, biosynthesis and content in plant tissues, fate during and after processing, effect on the human organism and interaction with the gut microbiota. Crit. Rev. Food Sci. Nutr..

[2] Royce S.G., Licciardi P.V., Beh R.C., Bourke J.E., Donovan C., Hung A.N., Khurana I., Liang J.L.J., Maxwell S., Mazarakis N. (2022). Sulforaphane prevents and reverses allergic airways disease in mice via anti-inflammatory, antioxidant, and epigenetic mechanisms. Cell. Mol. Life Sci..

[3] Li J., Wang J., Zhang N., Li Y., Cai Z., Li G., Liu Z., Liu Z., Wang Y., Shao X. (2023). Anti-aging activity and their mechanisms of natural food-derived peptides: Current advancements. Food Innov. Adv..

[4] Fusari C.M., Ramirez D.A., Camargo A.B. (2019). Simplified analytical methodology for glucosinolate hydrolysis products: A miniaturized extraction technique and multivariate optimization. Anal. Methods.

[5] Franco P., Spinozzi S., Pagnotta E., Lazzeri L., Ugolini L., Camborata C., Roda A. (2016). Development of a liquid chromatography-electrospray ionization-tandem mass spectrometry method for the simultaneous analysis of intact glucosinolates and isothiocyanates in Brassicaceae seeds and functional foods. J. Chromatogr. A.

[6] Chiang W.C.K., Pusateri D.J., Leitz R.E.A. (1998). Gas chromatography mass spectrometry method for the determination of sulforaphane and sulforaphane nitrile in broccoli. J. Agric. Food Chem..

[7] (2019). Rapeseed and Rapeseed Meals—Determination of Glucosinolates Content—Method Using High-Performance Liquid Chromatography.

[8] Ares A.M., Valverde S., Bernal J.L., Nozal M.J., Bernal J. (2015). Development and validation of a LC-MS/MS method to determine sulforaphane in honey. Food Chem..

[9] (2020). Determination of Sulforphane in Brassica Napus-Liquid Chromatography-Tandem Mass Spectrometry.

[10] Liu Y., Zhang D., Li X., Xiao J., Guo L. (2022). Enhancement of ultrasound-assisted extraction of sulforaphane from broccoli seeds via the application of microwave pretreatment. Ultrason. Sonochem..

[11] Saylan M., Demirel R., Ayyildiz M.F., Chormey D.S., Cetin G., Bakirdere S. (2022). Nickel hydroxide nanoflower-based dispersive solid-phase extraction of copper from water matrix. Environ. Monit. Assess..

[12] Farajzadeh M.A., Pasandi S., Mohebbi A., Afshar Mogaddam M.R. (2022). Application of magnetic iron (III) oxinate nanocomposite as an efficient sorbent in magnetic dispersive solid phase extraction of pesticides. Microchem. J..

[13] Liu X.L., Li Y., Chen Z.S., Yang H., Cai Y.W., Wang S.H., Chen J.R., Hu B.W., Huang Q.F., Shen C. (2023). Advanced porous nanomaterials as superior adsorbents for environmental pollutants removal from aqueous solutions. Crit. Rev. Environ. Sci. Technol..

[14] Xiao Y., Ahmad T., Belwal T., Aadil R.M., Siddique M., Pang L., Xu Y. (2023). A review on protein based nanocarriers for polyphenols: Interaction and stabilization mechanisms. Food Innov. Adv..

[15] Javed M., Matloob A., Ettoumi F.-e., Sheikh A.R., Zhang R., Xu Y. (2023). Novel nanobubble technology in food science: Application and mechanism. Food Innov. Adv..

[16] He J., Luo B., Zhang H., Li Z., Zhu N., Lan F., Wu Y. (2022). Surfactant-free synthesis of covalent organic framework nanospheres in water at room temperature. J. Colloid Interface Sci..

[17] Gao M., Deng L., Kang X., Fu Q., Zhang K., Wang M., Xia Z., Gao D. (2020). Core-shell structured magnetic covalent organic frameworks for magnetic solid-phase extraction of diphenylamine and its analogs. J. Chromatogr. A.

[18] Yang S., Wang Y., He J., Yang R., Ma X., Yuan Y., Yue T., Sheng Q. (2023). Functionalized magnetic covalent organic framework nanocomposites for high-efficiency adsorption of ethyl carbamate from liquor. Food Front..

[19] Wang J.X., Li J., Gao M.X., Zhang X.M. (2018). Recent advances in covalent organic frameworks for separation and analysis of complex samples. Trac -Trends Anal. Chem..

[20] Zhao H., Wang L., Liu G., Liu Y., Zhang S., Wang L., Zheng X., Zhou L., Gao J., Shi J. (2023). Hollow Rh-COF@COF S-Scheme Heterojunction for Photocatalytic Nicotinamide Cofactor Regeneration. ACS Catal..

[21] Fan H., Mundstock A., Feldhoff A., Knebel A., Gu J., Meng H., Caro J. (2018). Covalent Organic Framework-Covalent Organic Framework Bilayer Membranes for Highly Selective Gas Separation. J. Am. Chem. Soc..

[22] Gao Y., Gao M., Chen G., Tian M., Zhai R., Huang X., Xu X., Liu G., Xu D. (2021). Facile synthesis of covalent organic frameworks functionalized with graphene hydrogel for effectively extracting organophosphorus pesticides from vegetables. Food Chem..

[23] Yan Y., Lu Y., Wang B., Gao Y., Zhao L., Liang H., Wu D. (2018). Self-Assembling Hydrophilic Magnetic Covalent Organic Framework Nanospheres as a Novel Matrix for Phthalate Ester Recognition. ACS Appl. Mater. Interfaces.

[24] Gao Y., Zhao C., Tan Q., Gao M., Chen G., Zhai R., Huang X., Xu X., Liu G., Wang J. (2022). Ternary magnetic Fe_3_O_4_@C_3_N_4_@covalent organic framework for facile extraction and determination of organophosphorus pesticides in fruit. Microchem. J..

[25] Kokotou M.G., Revelou P.K., Pappas C., Constantinou-Kokotou V. (2017). High resolution mass spectrometry studies of sulforaphane and indole-3-carbinol in broccoli. Food Chem..

[26] Chen R., Qiao X., Liu F., Chen X. (2023). Amino acid ionic liquid-based magnetic dispersive solid-phase extraction for benzimidazole residue analysis in fruit juice and human serum based on theoretical screening. Food Chem..

[27] Food and Drug Administration (FDA), USA Guidance for Industry: Q2(R1) Validation of Analytical Procedures: Text and Methodology. https://www.fda.gov/regulatory-information/search-fda-guidance-documents/q2r1-validation-analytical-procedures-text-and-methodology-guidance-industry.

[28] Fang L., Qiu F. (2023). Determination of neurotoxic shellfish poisoning toxins in shellfish by liquid chromatography-tandem mass spectrometry coupled with dispersive solid phase extraction. Heliyon.

[29] Wang M., Gao M., Zhang K., Wang L., Wang W., Fu Q., Xia Z., Gao D. (2019). Magnetic covalent organic frameworks with core-shell structure as sorbents for solid phase extraction of fluoroquinolones, and their quantitation by HPLC. Mikrochim. Acta.

[30] Shkinev V., Maksimova V., Mokhodoeva O., Larichev V., Spivakov B., Osmolovskaya O., Egorova A., Smirnova I., Dzhenloda R. (2022). Nanosized magnetite modified with poly(ethylene glycol) for efficient sorption of L-lysine-α-oxidase from the culture fluid. Mater. Lett..

[31] Liu G., Huang X., Lu M., Li L., Li T., Xu D. (2019). Facile synthesis of magnetic zinc metal-organic framework for extraction of nitrogen-containing heterocyclic fungicides from lettuce vegetable samples. J. Sep. Sci..

[32] Albalawi A.E., Khalaf A.K., Alyousif M.S., Alanazi A.D., Baharvand P., Shakibaie M., Mahmoudvand H. (2021). Fe3O4(@)piroctone olamine magnetic nanoparticles: Synthesize and therapeutic potential in cutaneous leishmaniasis. Biomed. Pharmacother..

[33] Guo H., Li Y., Li Y., He X., Chen L., Zhang Y. (2023). Construction of Stable Magnetic Vinylene-Linked Covalent Organic Frameworks for Efficient Extraction of Benzimidazole Fungicides. ACS Appl. Mater. Interfaces.

[34] Zhang J., Li X.Q., Ge P., Zhang B., Wen L., Gu C.H., Zhou X. (2022). Sulforaphene: Formation, Stability, Separation, Purification, Determination and Biological Activities. Sep. Purif. Rev..

[35] Wu Y., Mao J., You Y., Liu S. (2014). Study on degradation kinetics of sulforaphane in broccoli extract. Food Chem..

[36] Tan C., Li J., Liu W., Zhao Q., Wang X., Li Y. (2020). Core-shell magnetic covalent organic framework nanocomposites as an adsorbent for effervescent reaction-enhanced microextraction of endocrine disruptors in liquid matrices. Chem. Eng. J..

[37] Li Y.X., Zhang H.N., Chen Y.T., Huang L., Lin Z., Cai Z.W. (2019). Core-Shell Structured Magnetic Covalent Organic Framework Nanocomposites for Triclosan and Triclocarban Adsorption. ACS Appl. Mater. Interfaces.

[38] Almeida A.C.M., do Nascimento R.A., Amador I.C.B., Santos T.C.d.S., Martelli M.C., de Faria L.J.G., Ribeiro N.F.d.P. (2021). Chemically activated red mud: Assessing structural modifications and optimizing adsorption properties for hexavalent chromium. Colloids Surf. A Physicochem. Eng. Asp..

[39] Schwaab M., Steffani E., Barbosa-Coutinho E., Severo J.B. (2017). Critical analysis of adsorption/diffusion modelling as a function of time square root. Chem. Eng. Sci..

[40] Ozer A., Akkaya G., Turabik M. (2005). The biosorption of Acid Red 337 and Acid Blue 324 on Enteromorpha prolifera: The application of nonlinear regression analysis to dye biosorption. Chem. Eng. J..

[41] Fu D., Zhang Y.H., Lv F.Z., Chu P.K., Shang J.W. (2012). Removal of organic materials from TNT red water by Bamboo Charcoal adsorption. Chem. Eng. J..

[42] Wang Q., Zhao Y., Shi Z., Sun X., Bu T., Zhang C., Mao Z., Li X., Wang L. (2021). Magnetic amino-functionalized-MOF(M = Fe, Ti, Zr)@COFs with superior biocompatibility: Performance and mechanism on adsorption of azo dyes in soft drinks. Chem. Eng. J..

[43] Qin P., Chen D., Li D., Li M., Mu M., Gao Y., Zhu S., Lu M. (2023). Synthesis of spindle-like amino-modified Zn/Fe bimetallic metal-organic frameworks as sorbents for dispersive solid-phase extraction and preconcentration of phytohormoes in vegetable samples. Food Chem.

[44] Lima J.Z., Ferreira da Silva E., Patinha C., Duraes N., Vieira E.M., Rodrigues V.G.S. (2022). Sorption of arsenic by composts and biochars derived from the organic fraction of municipal solid wastes: Kinetic, isotherm and oral bioaccessibility study. Environ. Res..

[45] Zou C., Xu Z., Nie F., Guan K., Li J. (2023). Application of hydroxyapatite-modified carbonized rice husk for the adsorption of Cr(VI) from aqueous solution. J. Mol. Liq..

[46] De Oliveira C., Renda C.G., Moreira A.J., Pereira O.A.P., Pereira E.C., Freschi G.P.G., Bertholdo R. (2023). Evaluation of a graphitic porous carbon modified with iron oxides for atrazine environmental remediation in water by adsorption. Environ. Res..

[47] Barkakati P., Begum A., Das M.L., Rao P.G. (2010). Adsorptive separation of Ginsenoside from aqueous solution by polymeric resins: Equilibrium, kinetic and thermodynamic studies. Chem. Eng. J..

[48] Hassan R., Abo Eldahab H.M.M., Shehata F.A., El-Reefy S.A., Mohamed S.A. (2023). Proficiency of some synthetic alginate derivatives for sequestration of Iodine-131 from radioactive liquid waste. Environ. Technol..

[49] Zhao J., Dai Y. (2022). Tetracycline adsorption mechanisms by NaOH-modified biochar derived from waste Auricularia auricula dregs. Environ. Sci. Pollut. Res. Int..

[50] ZA A.L., Badjah A.Y., Alharbi O.M.L., Ali I. (2020). Copper carboxymethyl cellulose nanoparticles for efficient removal of tetracycline antibiotics in water. Environ. Sci. Pollut. Res. Int..

[51] Baggiani C., Giraudi G., Giovannoli C., Tozzi C., Anfossi L. (2004). Adsorption isotherms of a molecular imprinted polymer prepared in the presence of a polymerisable template. Anal. Chim. Acta.

[52] Chen R., Zhang X., Liu F., Liu C., Peng Q., Qiao X. (2022). Theoretical design and preparation of ionic liquid-based magnetic nanoparticles for the magnetic dispersive solid-phase extraction of benzimidazoles in human plasma. Sep. Purif. Technol..

[53] Hadavifar M., Bahramifar N., Younesi H., Li Q. (2014). Adsorption of mercury ions from synthetic and real wastewater aqueous solution by functionalized multi-walled carbon nanotube with both amino and thiolated groups. Chem. Eng. J..

[54] Guo Q.W., Ma Q.Q., Xue Z.H., Gao X.D., Chen H.X. (2018). Studies on the binding characteristics of three polysaccharides with different molecular weight and flavonoids from corn silk. Carbohydr. Polym..

[55] Guo X., Tian Y., Zhang M., Li Y., Wen R., Li X., Li X., Xue Y., Ma L., Xia C. (2018). Mechanistic Insight into Hydrogen-Bond- Controlled Crystallinity and Adsorption Property of Covalent Organic Frameworks from Flexible Building Blocks. Chem. Mater..

[56] Li Y., Wang C., Ma S., Zhang H., Ou J., Wei Y., Ye M. (2019). Fabrication of Hydrazone-Linked Covalent Organic Frameworks Using Alkyl Amine as Building Block for High Adsorption Capacity of Metal Ions. ACS Appl. Mater. Interfaces.

[57] Sangthong S., Weerapreeyakul N. (2016). Simultaneous quantification of sulforaphene and sulforaphane by reverse phase HPLC and their content in Raphanus sativus L. var. caudatus Alef extracts. Food Chem..

[58] Han D., Row K.H. (2011). Separation and Purification of Sulforaphane from Broccoli by Solid Phase Extraction. Int. J. Mol. Sci..

[59] Liang H., Yuan Q.P., Dong H.R., Liu Y.M. (2006). Determination of sulforaphane in broccoli and cabbage by high-performance liquid chromatography. J. Food Compos. Anal..

[60] Hafezian S.M., Azizi S.N., Biparva P., Bekhradnia A. (2019). High-efficiency purification of sulforaphane from the broccoli extract by nanostructured SBA-15 silica using solid-phase extraction method. J. Chromatogr. B Anal. Technol. Biomed Life Sci..

[61] Azizi S.N., Amiri-Besheli B., Sharifi-Mehr S. (2011). The Isolation and Determination of Sulforaphane from Broccoli Tissues by Reverse Phase-High Performance Liquid Chromatography. J. Chin. Chem. Soc..

[62] Campas-Baypoli O.N., Sanchez-Machado D.I., Bueno-Solano C., Ramirez-Wong B., Lopez-Cervantes J. (2010). HPLC method validation for measurement of sulforaphane level in broccoli by-products. Biomed. Chromatogr..

[63] Celik H., Ariburnu E., Baymak M.S., Yesilada E. (2014). A rapid validated HPLC method for determination of sulforaphane and glucoraphanin in broccoli and red cabbage prepared by various cooking techniques. Anal. Methods.

[64] Ares A.M., Bernal J., Martín M.T., Bernal J.L., Nozal M.J. (2013). Optimized Formation, Extraction, and Determination of Sulforaphane in Broccoli by Liquid Chromatography with Diode Array Detection. Food Anal. Methods.

