# Supplementary material for: Facile Preparation of Magnetic COF-on-COF for Rapid Adsorption and Determination of Sulforaphane from Cruciferous Vegetables
Source: Foods. 2024 Jan 26;13(3):409. doi: 10.3390/foods13030409 (PMC10855713; doi:10.3390/foods13030409)
Supplement: Supplementary file 1 [file foods-13-00409-s001.zip › foods-2830889-supplementary.pdf]

Supplementary Information

for

Facile preparation of magnetic COF-on-COF for rapid adsorption and determination of sulforaphane from cruciferous vegetables

Jie Zhou [1,†], Dan Xu [2,†], Jiayong Cao [3], Weiye Shi [4], Xuan Zhang [3], Huan Lin [1], Chen Yin [3], Lingyun Li [1], Donghui Xu [1] and Guangyang Liu [1,*]

1 State Key Laboratory of Vegetable Biobreeding, Institute of Vegetables and Flowers, Chinese Academy of Agricultural Sciences, Key Laboratory of Vegetables Quality and Safety Control, Ministry of Agri-culture and Rural Affairs of China, Beijing 100081, China; linhuan03@caas.cn (H.L.); xudonghui@caas.cn (D.X.)

2 College of Life Sciences, Yantai University, Yantai 264005, China

3 Hebei Key Laboratory of Quality and Safety Analysis-Testing for Agro-Products and Food, Hebei North University, Zhangjiakou 075000, China

4 Institute of Biological Science and Engineering, Hebei University of Science and Technology, Shijiazhuang 050018, China

* Correspondence: liuguangyang@caas.cn

† These authors contributed equally to this work.

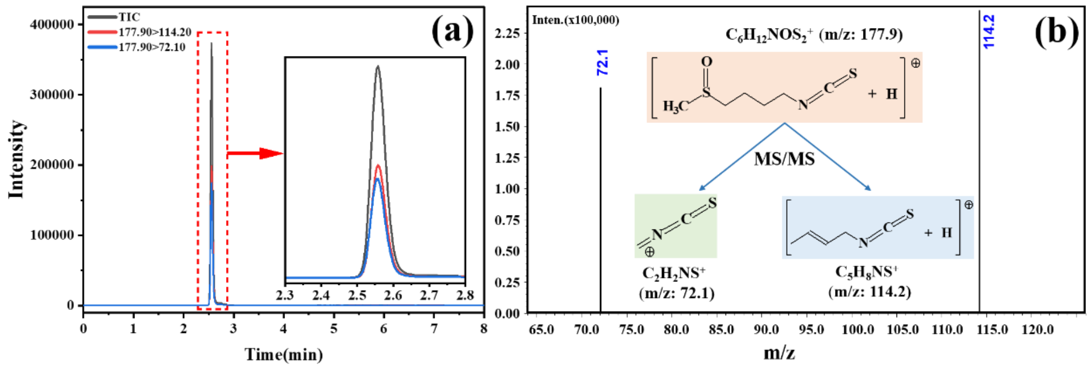

Figure S1. The ion chromatogram (a) and mass spectrum (b) of SFN in MeOH (0.01 mg/L)

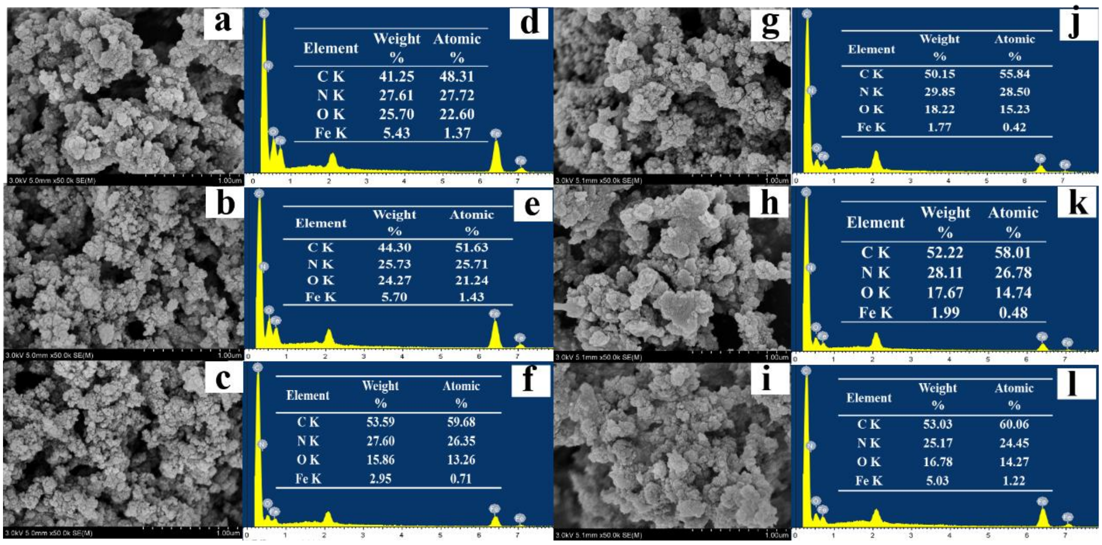

Figure S2. SEM (a-c, g-i) and EDS (d–f, j–l) results of Fe_3O_4@COFs-1 and MB-COFs (1–5), respectively.

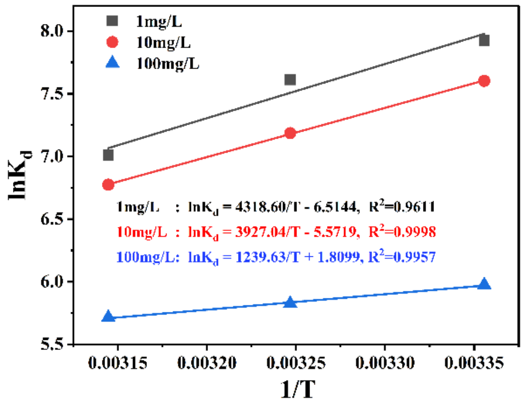

Figure S3. Vant Hoff's thermodynamic plot for adsorption of SFN.

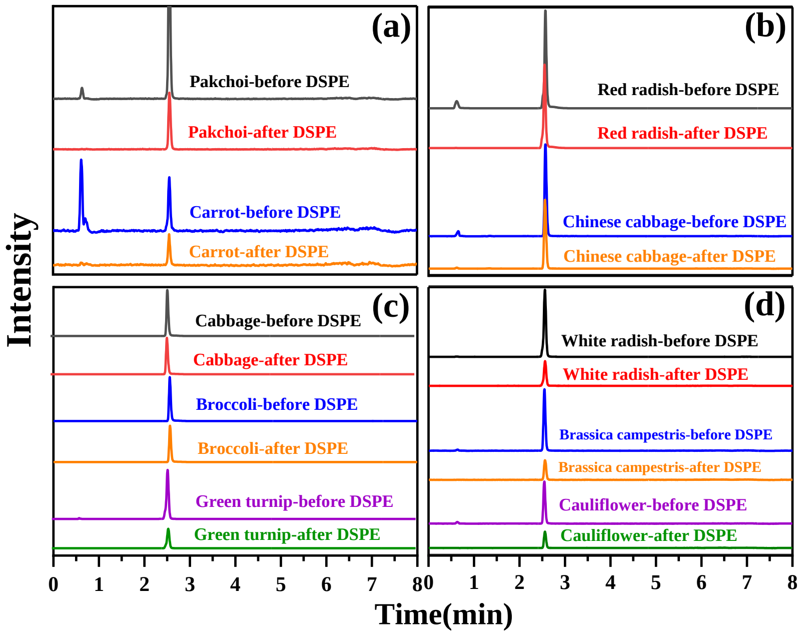

Figure S4. Extracted ion chromatograms of SFN in real cruciferous vegetable samples before and after MSPE. Pakchoi and carrot (a), red radish and Chinese cabbage (b), cabbage, broccoli and green turnip (c), and white radish, Brassica campestris, and cauliflower (d).

Table S1 The molecular information, ESI and MS/MS parameters of SFN.

**Parameters**	**Information / Value**	
**Molecular information**		
CAS number	4478-93-7	
Molecular formula	$C_6H_{11}NOS_2$	
Molecular weight (g/mol)	177.3	
Chemical structure	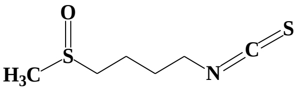	
**ESI parameters**		
Interface voltage (V)	3000	
Interface temperature (°C)	300	
DL temperature (°C)	250	
Heat block temperature (°C)	400	
Nebulizing gas flow (L/min)	3	
Heating gas flow (L/min)	10	
Driving gas flow (L/min)	10	
**MS/MS parameters**		
Retention time (min)	2.52	
Precursor (m/z)	177.9	
Product (m/z)	114.2*	72.1
Dwell time (ms)	100	100

Q1 Pre Bias (V)	-13	-13
Collision energy (V)	-12	-27
Q3 Pre Bias (V)	-21	-29

*Quantification ion.

Table S2 MB-COFs with different amounts of ligands and their adsorption efficiency for SFN with different concentrations (mg/L)

Serial No.	Adsorbents	Fe_3O_4@ COFs-1 (g)	TAPB (g)	MPA (g)	Adsorption efficiency (%)		
					0.5 mg/L	5 mg/L	50 mg/L
**1**	Fe_3O_4@COFs-1	0.5	0	0	86.87±0.34	83.94±0.34	83.06±0.11
**2**	MB-COFs-1	0.5	0.159	0.09	94.15±0.38	89.14±0.20	88.15±0.11
**3**	MB-COFs-2	0.5	0.2835	0.135	95.46±0.02	90.71±0.03	90.14±0.06
**4**	MB-COFs-3	0.5	0.318	0.18	95.49±0.14	91.42±0.10	90.63±0.12
**5**	MB-COFs-4	0.5	0.477	0.27	97.33±0.06	93.20±0.02	92.50±0.22
**6**	MB-COFs-5	0.5	0.636	0.36	97.28±0.02	93.22±0.08	92.37±0.06
**7**	COFs-1	/	/	/	79.69±1.23	63.08±8.75	55.01±0.54
**8**	COFs-2	0	0.477	0.27	35.00±2.17	21.15±1.22	31.52±0.71

Table S3 Surface area, pore volume and size of prepared samples

Samples	Surface Area (S_{BET}, m^2/g)	Pore Volume (V_{total}, cm^3/g)	BJH Pore Size (nm)
MB-COFs-4	40.5068	0.181975	16.5031
Fe3O4@COFs-1	61.6782	0.270135	15.231
COFs-1	63.5683	0.277305	19.6184
COFs-2	67.2413	0.311663	17.6443

Table S4 Parameters of kinetic models for SFN adsorption by MB-COFs-4.

Parameter of kinetic models	Values
$Q_{e, exp}$ (mg/g)	11.804
**Pseudo-first order**	
Q_e (mg/g)	2.457
k_1(min^{-1})	0.0251
R^2	0.7905
**Pseudo-second-order**	
Q_e (mg/g)	11.501
k_2 (g mg^{-1} min^{-1})	0.1067
R^2	0.9997
**Ritchie-second-order**	
Q_e (mg/g)	10.890
k_r (min^{-1})	3.707
R^2	0.9202
**Intra-particle diffusion**	

k_{i1} (mg g^{-1} $min^{-0.5}$)	2.166
C_1 (mg/g)	5.876
R_1^2	0.9987
k_{i2}(mg g^{-1} $min^{-0.5}$)	0.345
C_2 (mg/g)	9.091
R_2^2	0.9802
k_{i3} (mg g^{-1} $min^{-0.5}$)	0.1665
C_3 (mg/g)	9.974
R_3^2	0.9950
**Liquid-film diffusion**	
k_j (min^{-1})	0.0251
A (mg/g)	-1.570
R^2	0.7905
**Elovich model**	
α (mg·g^{-1}·min^{-1})	713.46
β (g mg^{-1})	1.333
R^2	0.9121
**Frusawa and Smith (F&S) model**	
$\beta_1 S$	0.00264
R^2	0.5790
**Mathews and Weber (M&W) model**	
$\beta_1 S$	0.00265
R^2	0.5788
**Boyd's model**	
R^2	0.7987

Table S5 SFN adsorption equilibrium parameters in MB-COFs-4 at different temperatures.

Isotherm models	Temperature (°C)			
	**35**	**45**	**55**	**65**
**Langmuir**				
Q_m (mg g^{-1})	14.728	15.480	16.103	15.576
K_L (L mg^{-1})	0.0415	0.0386	0.0337	0.0353
R_L	0.004-0.96	0.004-0.96	0.004-0.96	0.004-0.97
R^2	0.9956	0.9995	0.9968	0.9894
**Freundlich**				
K_F ($mg^{1-(1/n)}$·$L^{1/n}$·g^{-1})	1.8232	1.666	1.567	1.576
n	2.426	2.260	2.193	2.220
R^2	0.9508	0.9631	0.9778	0.9554
**Dubinin-Rabushkevich**				
Q_m (mg g^{-1})	10.796	10.993	10.958	10.933
B_D (mol^2 kJ^{-2})	14.187	13.747	13.567	14.001
E (kJ mol^{-1})	0.188	0.191	0.192	0.189
R^2	0.9209	0.8992	0.8879	0.9059
**Temkin**				

K_T (L mg^{-1})	0.435	0.370	0.331	0.339
b_T (kJ mol^{-1} L^{-1})	0.817	0.775	0.773	0.816
R^2	0.9907	0.9962	0.9979	0.9853
**Redlich-Peterson**				
a_R (mg L^{-1})	0.584	0.600	0.638	0.634
g	0.588	0.558	0.544	0.550
R^2	0.9752	0.9764	0.9843	0.9696

Table S6 SFN content in MB-COFs-4 in fresh cruciferous vegetables.

Sample	SFN content(mg/kg FW)
Cauliflower	0.49
Brassica campestris	0.09
Chinese cabbage	0.40
Carrot	0.01
Cabbage	3.84
Green turnip	6.10
White radish	3.88
Red radish	9.30
Pakchoi	0.35
Broccoli	36.78

The models for adsorption kinetics

To explore the kinetic behavior between MB-COFs and SFN, several kinetic models, including the pseudo-first-order (1), pseudo-second-order (2), and Ritchie-second-order (3), Webber-Morris's intra-particle diffusion (4), liquid-film diffusion (5), Elovich (6), Frusawa and Smith (F&S) (7), Mathews and Weber (M&W) (8) and Boyd's (9)[1] models were used to fit experimental data. The expressions of the kinetic models are as follows[2-7]:

$$\ln(Q_e - Q_t) = \ln Q_e - k_1 t \qquad (S1)$$

$$\frac{t}{Q_t} = \frac{1}{Q_e^2} k_2^{-1} + \frac{t}{Q_e} \qquad (S2)$$

$$\frac{1}{Q_t} = \frac{1}{K_r Q_e t} + \frac{1}{Q_e} \qquad (S3)$$

$$Q_t = K_i t^{0.5} + C \qquad (S4)$$

$$\ln(1 - \frac{Q_t}{Q_e}) = - k_j t + A \qquad (S5)$$

$$Q_t = \frac{1}{\beta}\ln(\alpha\beta) + \frac{1}{\beta}\ln t \quad (S6)$$

$$\ln\left(\frac{C_t}{C_0} - \frac{1}{1+mK_L}\right) = \ln\frac{mK_L}{1+mK_L} - \frac{1+mK_L}{mK_L}\beta_1 St \quad (S7)$$

$$\ln\frac{C_t}{C_0} = -\beta_1 St \quad (S8)$$

$$B_t = -\ln\frac{\pi^2}{6} - \ln\left(1 - \frac{Q_t}{Q_e}\right) \quad \text{for } \frac{Q_t}{Q_e} > 0.85 \quad (S9\text{-}1)$$

$$B_t = \left(\pi^{0.5} - \left(\pi - \frac{\pi^2}{3}\frac{Q_t}{Q_e}\right)^{0.5}\right)^2 \quad \text{for } \frac{Q_t}{Q_e} \leq 0.85 \quad (S9\text{-}2)$$

where Q_t and Q_e (mg/g) are the adsorption capacities of SFN at time t and at equilibrium, respectively; t (min) is the adsorption time. α and β are the initial rate constant (mg·g^{-1}·min^{-1}) and desorption constant (g mg^{-1}) in Elovich model, respectively. k_1 (min^{-1}), k_2 (g mg^{-1} min^{-1}), k_i (mg g^{-1} min$^{-0.5}$), k_j (min^{-1}), k_r (min^{-1}) and are the rate constants of these models. C_0 and C_t (mg/L) are the SFN concentrations at the initial and time t, respectively, m (g/L) is the mass of adsorbent per unit volume of particle free SFN solution, K_L (L/mg) is the Langmuir constant (obtained from Langmuir isotherm model), β_1 (cm/s) is the mass transfer coefficient and S is the outer surface of adsorbent per unit volume of particle free slurry (cm^{-1}). In general, the value of S is difficult to determine. Thus, the β_1S value was used to describe the adsorption process[4]. Boyd's plot was established by plotting the Boyd's function (B_t) against time(t), which is used to identify whether external transport controls the rate of adsorption.

The models for adsorption isotherm

To further investigate the adsorption isotherm of SFN by MB-COFs-4, Langmuir (10) [6], Freundlich (11) [6], Dubinin-Rabushkevich (D-R) (12) [8], Temkin (13) [8], and Redlich-Peterson (R-P) (14)[9] models were used to fit the experimental data. Among these models, the Langmuir model assumes that the process involves monolayer adsorption on homogenous sites with uniform energy levels within the adsorbent surface, while the Freundlich model assumes that the process is multilayer adsorption and occurs on the heterogeneous surface with a non-uniform energy level. The hybrid

R-P model combines the Freundlich and Langmuir models and may be used in both heterogeneous and homogeneous environments. The Temkin isotherm model has been developed taking into account the chemisorption between the adsorbate and adsorbent, which assumes that the adsorption heat of all the molecules in the layer reduces linearly with the increasing coverage owing to adsorbate-adsorbent interactions. The D-R isotherm assumes that the characteristics of the adsorption curves are related to porosity of the adsorbents and is utilized to determine whether the adsorption process is physical or chemical [8,10-12]. The linear equations of these models are as follows:

$$\frac{C_e}{Q_e} = \frac{1}{Q_m K_L} + \frac{C_e}{Q_m} \quad \text{(S10)}$$

$$\ln Q_e = \ln K_F + \frac{1}{n} \ln C_e \quad \text{(S11)}$$

$$\ln Q_e = \ln Q_m - B_D \left(RT\ln\left(1 + \frac{1}{Ce}\right)\right)^2 \quad \text{(S12)}$$

$$Q_e = \frac{RT}{b_T} \ln C_e + \frac{RT}{b_T} \ln K_T \quad \text{(S13)}$$

$$\ln\left(\frac{C_e}{Q_e}\right) = g\ln C_e + \ln a_R \quad \text{(S14)}$$

where C_e (mg/L) represents the SFN concentrations at equilibrium, Q_e and Q_m (mg/g) represent the equilibrium and maximum adsorption amount of SFN. K_L (L/mg) and K_F ($mg^{1-(1/n)}\cdot L^{1/n}\cdot g^{-1}$) represent the Langmuir and Freundlich adsorption constants, respectively. The separation factor (R_L, equal to $1/(1+C_0K_L)$) for Langmuir model is used to determine whether an adsorption system is favorable or unfavorable. $0<R_L<1$, $R_L>1$, $R_L=1$, and $R_L=0$ characterizes a favorable, unfavourable, linear and irreversible adsorption, respectively[4]. 1/n expresses a constant related to the surface heterogeneity and indicates different types of isotherms: $1/n = 0$ means the adsorption is irreversible, $0 < 1/n < 1$ means the favorable adsorption, and $1/n > 1$ means the opposite. B_D (mol^2 kJ^{-2}) is related to mean free energy E (kJ mol^{-1}) of the transfer of 1 mol of solute from infinity to the surface of adsorbent, with $E=1/(2B_D)^{0.5}$. K_T (L/mg) and b_T (kJ mol^{-1}) is equilibrium binding constant and the Temkin isotherm constant, respectively. a_R (mg L^{-1}) and g (dimensionless) are the R-P constants and the value of g must range between 0 and 1. T (K) is temperature and R (8.314×10^{-3} kJ mol^{-1} K^{-1}) is gas constant.

References

1. Almeida, A.C.M.; do Nascimento, R.A.; Amador, I.C.B.; Santos, T.C.d.S.; Martelli, M.C.; de Faria, L.J.G.; Ribeiro, N.F.d.P. Chemically activated red mud: assessing structural modifications and optimizing adsorption properties for hexavalent chromium. *Colloids and Surfaces A: Physicochemical and Engineering Aspects* **2021**, *628*, doi:10.1016/j.colsurfa.2021.127325.
2. Schwaab, M.; Steffani, E.; Barbosa-Coutinho, E.; Severo, J.B. Critical analysis of adsorption/diffusion modelling as a function of time square root. *Chemical Engineering Science* **2017**, *173*, 179-186, doi:10.1016/j.ces.2017.07.037.
3. Ozer, A.; Akkaya, G.; Turabik, M. The biosorption of Acid Red 337 and Acid Blue 324 on Enteromorpha prolifera: The application of nonlinear regression analysis to dye biosorption. *Chemical Engineering Journal* **2005**, *112*, 181-190, doi:10.1016/j.cej.2005.07.007.
4. Fu, D.; Zhang, Y.H.; Lv, F.Z.; Chu, P.K.; Shang, J.W. Removal of organic materials from TNT red water by Bamboo Charcoal adsorption. *Chemical Engineering Journal* **2012**, *193*, 39-49, doi:10.1016/j.cej.2012.03.039.
5. Wang, Q.; Zhao, Y.; Shi, Z.; Sun, X.; Bu, T.; Zhang, C.; Mao, Z.; Li, X.; Wang, L. Magnetic amino-functionalized-MOF(M = Fe, Ti, Zr)@COFs with superior biocompatibility: Performance and mechanism on adsorption of azo dyes in soft drinks. *Chemical Engineering Journal* **2021**, *420*, doi:10.1016/j.cej.2021.129955.
6. Qin, P.; Chen, D.; Li, D.; Li, M.; Mu, M.; Gao, Y.; Zhu, S.; Lu, M. Synthesis of spindle-like amino-modified Zn/Fe bimetallic metal-organic frameworks as sorbents for dispersive solid-phase extraction and preconcentration of phytohormoes in vegetable samples. *Food Chem* **2023**, *409*, 135272, doi:10.1016/j.foodchem.2022.135272.
7. Lima, J.Z.; Ferreira da Silva, E.; Patinha, C.; Duraes, N.; Vieira, E.M.; Rodrigues, V.G.S. Sorption of arsenic by composts and biochars derived from the organic fraction of municipal solid wastes: Kinetic, isotherm and oral bioaccessibility study. *Environ Res* **2022**, *204*, 111988, doi:10.1016/j.envres.2021.111988.
8. Barkakati, P.; Begum, A.; Das, M.L.; Rao, P.G. Adsorptive separation of Ginsenoside from aqueous solution by polymeric resins: Equilibrium, kinetic and thermodynamic studies. *Chemical Engineering Journal* **2010**, *161*, 34-45, doi:10.1016/j.cej.2010.04.018.
9. Hassan, R.; Abo Eldahab, H.M.M.; Shehata, F.A.; El-Reefy, S.A.; Mohamed, S.A. Proficiency of some synthetic alginate derivatives for sequestration of Iodine-131 from radioactive liquid waste. *Environ Technol* **2023**, 1-14, doi:10.1080/09593330.2023.2213447.
10. Zhao, J.; Dai, Y. Tetracycline adsorption mechanisms by NaOH-modified biochar derived from waste Auricularia auricula dregs. *Environ Sci Pollut Res Int* **2022**, *29*, 9142-9152, doi:10.1007/s11356-021-16329-5.
11. de Oliveira, C.; Renda, C.G.; Moreira, A.J.; Pereira, O.A.P.; Pereira, E.C.; Freschi, G.P.G.; Bertholdo, R. Evaluation of a graphitic porous carbon modified with iron oxides for atrazine environmental remediation in water by adsorption. *Environ Res* **2023**, *219*, 115054, doi:10.1016/j.envres.2022.115054.
12. ZA, A.L.; Badjah, A.Y.; Alharbi, O.M.L.; Ali, I. Copper carboxymethyl cellulose nanoparticles for efficient removal of tetracycline antibiotics in water. *Environ Sci Pollut Res Int* **2020**, *27*, 42960-42968, doi:10.1007/s11356-020-10189-1.
